# Can foliar application of soluble monoammonium phosphate effectively alleviate herbicide-induced oxidative stress in key crops?

**DOI:** 10.3389/fpls.2025.1504244

**Published:** 2025-02-28

**Authors:** Josiane Viveiros, Luiz Gustavo Moretti, Israel Alves Filho, Marcela Pacola, Lucas Moraes Jacomassi, Vitor Alves Rodrigues, Amine Jamal, João William Bossolani, José Roberto Portugal, Caio Antonio Carbonari, Carlos Alexandre Costa Crusciol

**Affiliations:** ^1^ Department of Crop Science, School of Agricultural Sciences (FCA), Sao Paulo State University (UNESP), Botucatu, Brazil; ^2^ Office Chérifien des Phosphates (OCP), OCP Nutricrops, Casablanca, Morocco; ^3^ Plant Protection Department, School of Agricultural Sciences (FCA), Sao Paulo State University (UNESP), Botucatu, Brazil

**Keywords:** carfentrazone-ethyl, soluble monoammonium phosphate, oxidative stress, nitrogen, phosphorus, photosynthesis

## Abstract

Phosphorus (P) and nitrogen (N) directly impact final crop productivity by playing essential roles in photosynthesis, ATP formation, carbon assimilation, cell division, and transport. Compared with nutrient application to soil, the nutrients are applied directly to leaves provides a faster response because the nutrients enter plant metabolism more quickly. Foliar fertilization with nutritional supplements can intend to increase crop yields, and little is known about its ability to reduce oxidative stress. This study evaluated the effects of foliar fertilization on crop recovery from phytotoxicity induced by herbicide exposure. Phytotoxicity was induced in soybean, maize, and cotton plants by applying the herbicide carfentrazone-ethyl (at V_3_, V_3_ and V_4_ growth stages, respectively), which induces the accumulation of reactive oxygen species in the cytoplasm, leading to membrane rupture and the appearance of chlorotic spots on leaves. Phytotoxicity induction was followed by the foliar application of monoammonium phosphate (MAP) as a source of N and P. Leaf nutrient content, gas exchange performance, pigment content, photosynthetic enzyme activity, antioxidant metabolism, oxidative stress, proline content, metabolite content, and biometric parameters were evaluated. MAP supplementation increased chlorophyll content, and RuBisCO activity by up to 20.5% (maize) and 16.2% (cotton), respectively, resulting in higher net photosynthetic rates (26.3%; cotton), stomatal conductance (45.7%; cotton), water use efficiency (35.6%; cotton), and carboxylation efficiency (45%; cotton). The activities of antioxidant enzymes also increased, and the concentrations of oxidative stress indicators decreased (H_2_O_2_: 33.7% and MDA: 28.3%; soybean). Furthermore, the productivity of all three crops increased, suggesting that foliar application of MAP is an efficient strategy for attenuating phytotoxicity symptoms in crops.

## Highlights

- Foliar application of soluble MAP improves gas exchange and antioxidant parameters, suggesting mitigation of phytotoxicity.
- Foliar application of soluble MAP significantly increases chlorophyll content and RuBisCO activity.
- Targeted nutrient supplementation enables crop recovery from phytotoxicity while also increasing productivity.

## 1 Introduction

Soybean, cotton, and maize are essential crops in tropical agriculture due to their economic significance, agronomic adaptability, and contribution to sustainable farming systems (Momesso et al., 2022). Soybean serves as a primary protein and oil source, cotton thrives in warm climates with high drought tolerance, and maize is vital for food security and crop rotation (Viveiros et al., 2024). Foliar application of nutrients and biostimulants enhances crop performance by improving nutrient assimilation, particularly under restricted root absorption (Moreira et al., 2022). The integration of foliar spraying with nutrients, biostimulants, and other agrochemicals in these crops has been shown to enhance physiological responses, mitigate stress-induced yield losses, and improve overall crop productivity (Rodrigues et al., 2021a). Given their extensive cultivation and contribution to global agricultural output, optimizing foliar application strategies supports sustainable intensification, ensuring greater resilience and efficiency in tropical agroecosystems (Momesso et al., 2019).

Weeds compete with crops both nutritionally and physically (Priess et al., 2020). Herbicides are the easy-to-apply and cost-effective form of weeds control (Tataridas et al., 2022; Ofosu et al., 2023), its improper use can cause environmental contamination and toxicity to humans and non-target plants (Mehdizadeh et al., 2021; Van Bruggen et al., 2021). Moreover, the repeated use of or reliance on herbicides with a single mode of action can lead to the selection of resistant weed species, which are more difficult to control and require the use of higher doses or alternative herbicides, worsening environmental and health risks (Gupta, 2018; Song et al., 2020). The use of herbicides is further complicated by the potential for drift, which is influenced by wind speed, temperature, and relative humidity (Langaro et al., 2017). If adjacent crops are not tolerant to the applied herbicide, contact with the product due to drift may result in phytotoxic stress (Hand et al., 2021).

Carfentrazone-ethyl is a contact herbicide that inhibits protoporphyrinogen oxidase (PPOX), an enzyme responsible for converting protoporphyrinogen IX into the chlorophyll precursor protoporphyrin IX in the chloroplast (Sherman et al., 1991; Bertucci et al., 2019). Its inhibition leads to the accumulation of protoporphyrinogen IX, which diffuses into the cytoplasm and undergoes nonenzymatic oxidation to protoporphyrin IX, disrupting cellular function (Dayan et al., 1997). In the presence of light, cytoplasmic protoporphyrin IX forms singlet oxygen (^1^O_2_), initiating the process of lipid peroxidation (Moretti et al., 2021). Within two days of carfentrazone-ethyl exposure, protein and lipid oxidation lead to the loss of chlorophyll and carotenoids, as well as membrane rupture. The most affected lipids are phospholipids, which form the lipid bilayer of cellular membranes, particularly in chloroplasts, where photosynthesis takes place. Among the oxidized proteins, key components of the photosynthetic apparatus are affected, including proteins from the photosystem II complex (Farooq et al., 2013, 2019). Contact herbicides such as carfentrazone-ethyl applied to weeds that have emerged before soybean, maize and cotton crops planting or applied in a targeted manner after crops emergence. This latter usage raises the possibility of phytotoxicity induction due to drift (Li et al., 2022; Sahu et al., 2023).

Phytotoxicity can increase the crops production of reactive oxygen species (ROS), such as superoxide anion (O_2_^-^), hydrogen peroxide (H_2_O_2_), hydroxyl radical (OH^-^), and ^1^O_2_ (Farmer and Mueller, 2013). ROS production is a normal part of plant development, but excess ROS production under stress conditions disrupts the redox balance that regulates the plant’s defense system, leading to oxidative stress (Farooq et al., 2019; Silva et al., 2020). The defense system of plants includes both enzymes, such as superoxide dismutase (SOD), catalase (CAT) and ascorbate peroxidase (APX), and non-enzymatic molecules, including ascorbic acid, vitamin E, flavonoids, proline, and glutathione (Davar et al., 2013). All of these molecules require the presence of nutrients that directly participate in plant metabolism and membrane integrity (Adrees et al., 2015).

Nitrogen (N) and phosphorus (P) are essential macronutrients that support plant growth, metabolism, and stress tolerance by contributing to energy transfer, photosynthesis, and protein synthesis. Their availability is critical under stress conditions, as they activate the antioxidant defense system to mitigate oxidative damage from ROS (Moreira et al., 2016, 2017, 2018). Plants with an adequate nutrient supply tend to have greater stress tolerance (Marschner, 2012). Although crops can obtain nutrients from soil, that may be a necessary supplement during periods of high nutrient demand (Fernández and Brown, 2013). Foliar application provides a faster response than soil application because nutrients taken up by leaves directly enter metabolic processes (Oliveira et al., 2022). The foliar application of nutrients to plants in the vegetative stage can help protect the photosynthetic system and activate plant antioxidant defense systems, thereby reducing symptoms of stress (Reid et al., 1997; Taiz et al., 2017). However, nutrient absorption and, consequently, the efficiency of foliar application vary according to the nutrient, plant, environment, and the specific product applied (Fernández and Brown, 2013).

The objective of this study was to evaluate the efficacy of foliar application of soluble monoammonium phosphate (MAP) in mitigating herbicide-induced oxidative stress in soybean, maize, and cotton crops. Specifically, we evaluated the effects of MAP supplementation on physiological and biochemical parameters such as chlorophyll content, photosynthetic enzyme activity, antioxidant metabolism, and oxidative stress indicators to determine whether this practice can enhance crops productivity by reducing the phytotoxic effects of the herbicide carfentrazone-ethyl.

## 2 Materials and methods

### 2.1 Location descriptions

The study encompassed the 2020/2021 and 2021/2022 growing seasons of soybean (between the months of October and March) and cotton (between the months of December and June) and the 2021 and 2022 growing seasons of maize (between the months of February and June). Each crop was grown in a different location in the state of São Paolo, Brazil: soybean at the Lageado Experimental Farm in Botucatu, maize in Santa Cruz do Rio Pardo, and cotton in Riolândia.

Lageado Experimental Farm, Botucatu (soybean): This site belongs to the Faculty of Agricultural Sciences of São Paulo State University “Júlio de Mesquita Filho” and is located at 22° 83′ 3″ S, 48° 42′ 64″ W, 765 m above sea level (m.a.s.l.). The regional climate is Cwa, which corresponds to hot, humid summers and dry winters (Alvares et al., 2013). The average annual temperature and precipitation are approximately 22°C and 1360 mm, respectively (Unicamp, 2019).

Santa Cruz do Rio Pardo (maize): This site is located at 22° 50′ 7″ S, 49° 31′ 09.4″ W, 467 m.a.s.l. The regional climate is Cwa, and the temperature rarely drops below 11°C. The average temperature range is 15 to 31°C, and the annual precipitation is approximately 1236.5 mm.

Piapara Farm, Riolândia (cotton): This site is located at 19° 56′ 36.9″ S, 49° 37′ 25.4″ W, 438 m.a.s.l. The regional climate is Cwa. Temperatures range between 12°C to 33°C, and the annual precipitation is approximately 1221 mm.

**Figure 1:**
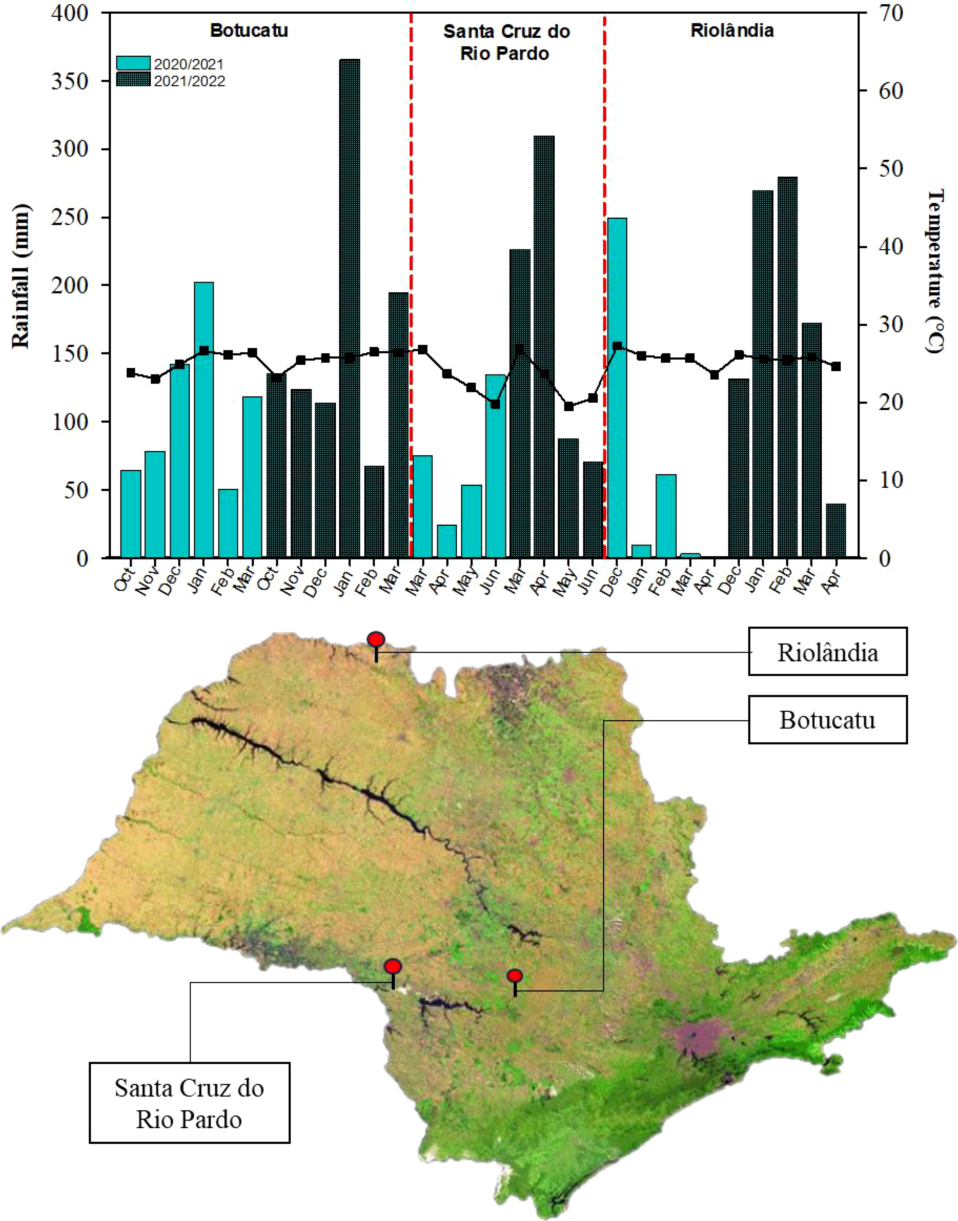
Average monthly temperatures and precipitation (mm) during the first and second growing season of soybean in Botucatu, maize in Santa Cruz do Rio Pardo, and cotton in Riolândia. The map shows the locations of each of the three municipalities in the state of São Paulo.

**Figure 1** presents the monthly average temperature and precipitation at the locations during the study period. The soil at each location was analyzed after the harvest in 2021, and the results are presented in **Table 1**.

**Table 1:** Soil characteristics at a depth of 0–20 cm prior to the 2021 growing season at the three study locations.

SOIL CLASSIFICATION	Site 1 Soybean	Site 2 Maize	Site 3 Cotton
	Oxisols		
Climate (Köppen-Geiger)	Cwa		
pH (CaCl_2_)	4.7	5.1	5.2
MO (g dm^-3^)	24	30	26
P (mg dm^-3^)	27	31	22
S (mg dm^-3^)	17	18	13
Al^+3^ (mmol dm^-3^)	0	0	0
H+Al^+3^ (mmol dm^-3^)	28	42	25
K (mmol dm^-3^)	3.5	3.8	3.1
Ca (mmol dm^-3^)	35	27	54
Mg (mmol dm^-3^)	14	12	11
SB* (mmol dm^-3^)	43	46.8	60
CEC** (mmol dm^-3^)	62	34	85
BS*** (%)	63	51	67
M**** (%)	0	0	0
Fe (mg dm^-3^)	15	20	21
Cu (mg dm^-3^)	1.6	1.7	10.4
Mn (mg dm^-3^)	14.0	8.4	12.0
Zn (mg dm^-3^)	3.0	9.6	2.5
B (mg dm^-3^)	0.6	0.34	0.42

*Sum of Bases; **Cation Exchange Capacity:

***Base Saturation. ****Aluminum Saturation.

**Figure 2:**
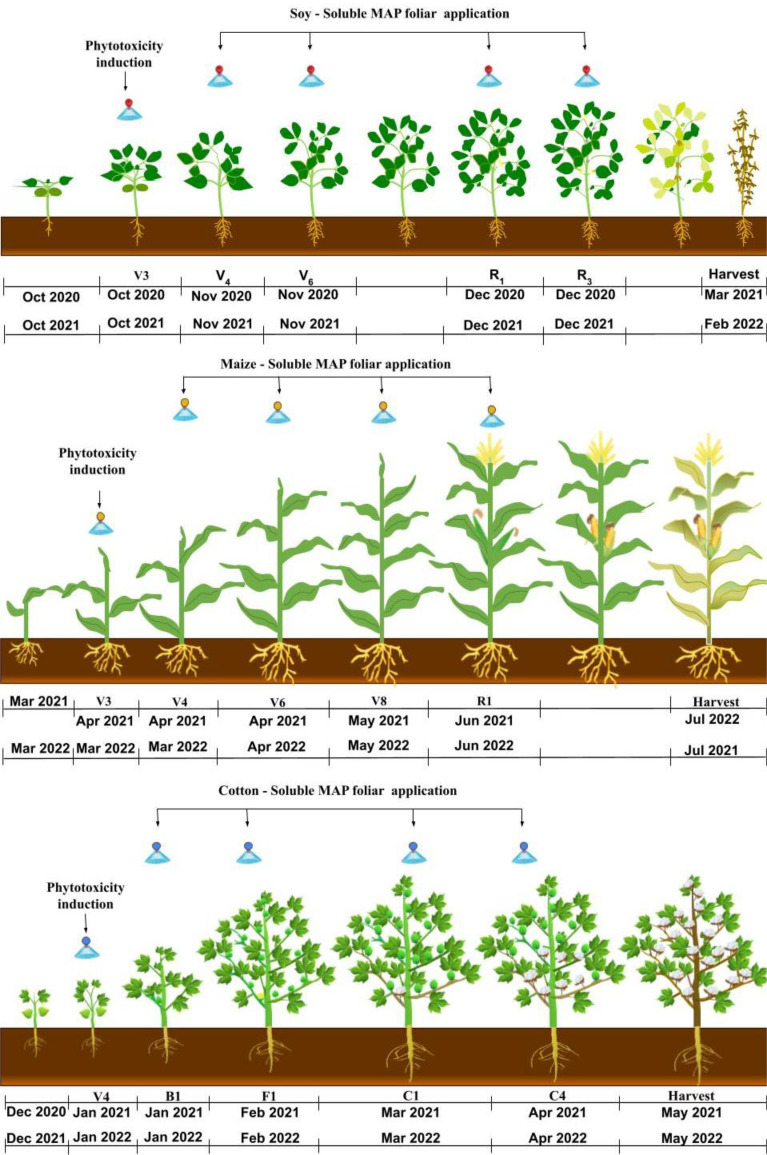
Timing of phytotoxicity induction and foliar application of soluble MAP during the soybean, maize, and cotton growing seasons.

### 2.2 Experimental design

The experiments were conducted in a randomized block design with three crops, seven treatments and four replications, that is, three experiments with a total of 28 plots. In the soybean experiment (Botucatu - SP), each plot contained seven 11-m-long rows with an inter-row spacing of 0.50 m (11 m long × 3 m wide, totaling 33 m^2^ per plot). In the maize experiment (Santa Cruz do Rio Pardo), each plot contained seven 11-m-long rows with an inter-row spacing of 0.45 m (11 m long × 2.70 m wide, totaling 29.7 m^2^ per plot). In the cotton experiment, each plot contained four 7-m-long rows with an inter-row spacing of 0.90 m (7 m long × 2.70 m wide, totaling 18.9 m^2^ per plot).

The seven treatments included two controls: an absolute control (Ac) with no application of carfentrazone-ethyl or MAP and a phytotoxicity control (Pc) with application of carfentrazone-ethyl but no application of MAP. Four of the treatments comprised phytotoxicity induction plus the application of MAP at a single specific growth stage (labeled according to the growth stage; see **Table 2**). The final treatment (All Ps) included phytotoxicity induction and the application of MAP at four growth stages.

**Table 2:** Experimental treatments.

TREATMENT	Phytotoxicity Induction	MAP Application
Soybean		
Absolute control (Ac)	None	None
Phytotoxicity control (Pc)	V_3_	None
V_4_	V_3_	V_4_
V_6_	V_3_	V_6_
R_1_	V_3_	R_1_
R_3_	V_3_	R_3_
All Phases (All Ps)	V_3_	V_4_+V_6_+R_1_+R_3_
Maize		
Absolute control (Ac)	None	None
Phytotoxicity control (Pc)	V_3_	None
V_4_	V_3_	V_4_
V_6_	V_3_	V_6_
V_8_	V_3_	V_8_
R_1_	V_3_	R_1_
All Phases (All Ps)	V_3_	V_4_+V_6_+V_8_+R_1_
Cotton		
Absolute control (Ac)	None	None
Phytotoxicity control (Pc)	V_4_	None
B_1_	V_4_	B_1_
F_1_	V_4_	F_1_
C_1_	V_4_	C_1_
C_4_	V_4_	C_4_
All Phases (All Ps)	V_4_	B_1_+F_1_+C_1_+C_4_

### 2.3 Application of treatments

The growth stage at which phytotoxicity was induced was selected based on the sensitivity of each crop: V_3_ for soybean (Fehr and Caviness, 1977) and maize (Ritchie et al., 1993) and V_4_ for cotton (Marur and Ruano, 2004). These growth stages are critical developmental phases in which plants are actively expanding their leaves and beginning to form essential structures for photosynthesis and nutrient assimilation. Focusing on these early stages ensured that the response to MAP was evaluated at a crucial moment for the formation of the photosynthetic apparatus and antioxidant pathways, maximizing the relevance of the final productivity results.

**Figure 3:**
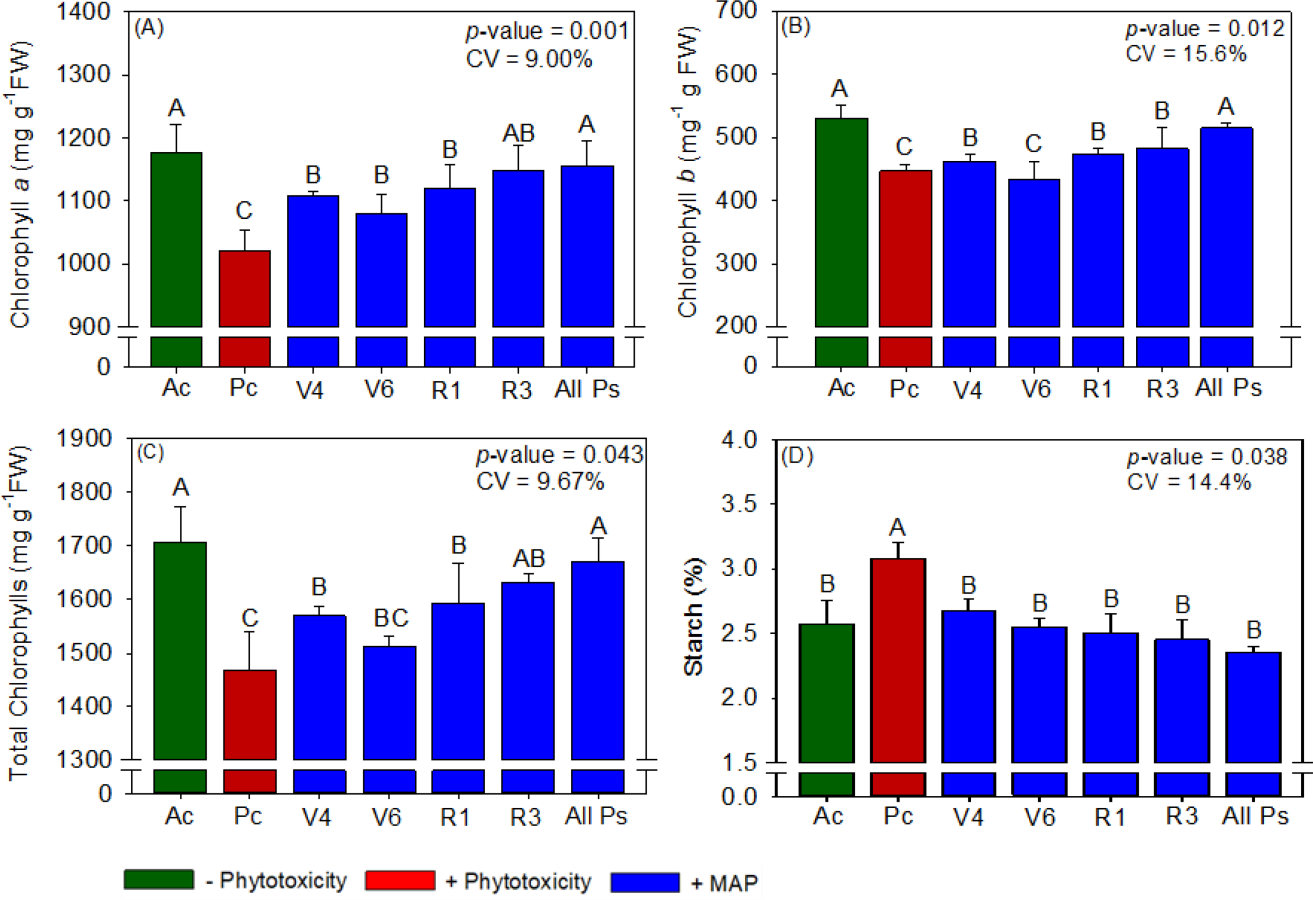
Response of various parameters — **(A)** chlorophyll *a*, **(B)** chlorophyll *b*, **(C)** total chlorophyll and **(D)** starch — as a function of foliar soluble MAP application in soybean leaves. Bars for the same crop with different letters are significantly different by Fisher’s protected least significant difference (LSD) test at *p* ≤ 0.05. Growing seasons were considered random effects.

Phytotoxicity was induced by applying carfentrazone-ethyl to soybean and cotton at a dose of 7 mL active ingredient ha^-^¹ + 0.5% mineral oil and to maize at a dose of 50 mL active ingredient ha^-^¹ + 0.5% mineral oil (Christoffoleti et al., 2002). These herbicide doses were selected after testing to determine the appropriate dose for inducing moderate leaf damage without compromising plant viability. The dose that resulted in visible damage to the leaf area but did not cause the death of plant was selected for each crop. This level of phytotoxicity facilitated the evaluation of the effectiveness of foliar MAP application in the recovery of the damaged plants. Foliar application of soluble MAP (12-61-00; Nutridrop^®^; OCP Morocco) as a source of P and N was performed at a dose of 5 kg ha^-^¹, equivalent to 0.55 kg ha^-1^ NH_4_^+^ and 3.05 kg ha^-1^ P_2_O_5_. **Table 2**, **Figures 2** and **3** describe the treatments and the timing of each application.

All spraying, both herbicide and MAP, was carried out with a constant pressure (CO_2_) backpack sprayer equipped with a 3-m-long boom with 6 fan nozzles (AXI 11002) spaced at intervals of 0.50 m. The spray volume and pressure were 150 L ha^-1^ and 1.80 bar, respectively.

### 2.4 Crop management practices

Soybean cultivar NEO 580 IPRO was planted at 16 plants m^-1^. Before planting, the seeds were treated with the fungicides carboxin + Tyrant® (100 g + 100 g active ingredient/100 kg seeds^-^¹) and a liquid inoculant containing *Bradyrhizobium japonicum* (Moretti et al., 2018, 2020, 2024). Base fertilization was carried out with 200 kg of granular MAP (11-52-00) applied to the sowing furrow and 70 kg of potassium applied to the soil surface.

Maize hybrid P3707VYH DuPont Pioneer was planted at 3 seeds m^-^¹. Before planting, the seeds were treated with the fungicides carboxin + Tyrant® (100 g + 100 g active ingredient/100 seeds^-^¹). The maize plants were fertilized with 280 kg ha^-^¹ of 28-08-16 in the sowing furrow. At stage V_4_, 172 kg ha^-^¹ of urea and 25 kg ha^-^¹ potassium were applied to the soil surface.

Cotton cultivar TMG 81 was planted at 9 plants m^-^¹. The seeds were treated with the fungicides carboxin + Tyrant® (100 g + 100 g active ingredient/100 kg seeds^-^¹), and the cotton plants were fertilized with 310 kg^-^¹ of 20-08-20 applied in the seeding furrow. At the beginning of stage V_4_, 204 kg of urea was applied to the soil surface.

### 2.5 Leaf analyses

For nutritional analysis and evaluations of oxidative stress, antioxidant metabolism, proline content, gas exchange, photosynthetic pigment content, RuBisCO activity, and metabolite content, leaf samples were collected from soybean plants at phenological stage R_4_ (fully developed pods), maize plants at stage R_2_ (white grains with a water bubble appearance), and cotton plants at stage C_5_ (opening of the first boll on the 5^th^ branch).

### 2.6 Nutritional analysis

Complete nutritional sampling of macro- and micronutrients was performed for each crop. For soybean, the third trefoil was collected from 10 plants, resulting in a total of 30 leaves (with petioles) per plot. For maize, the first leaf below the first ear was collected from 10 plants per plot, and the middle third of each leaf was used. For cotton, the third leaf, counting from the apex to the base, was collected from 10 plants per plot. After collection, the leaves were dried in an oven with forced-air circulation at 65°C for 72 h. The material was then ground in a Wiley mill on a sieve with a mesh diameter of 1 mm, and nutritional content was determined according to the methodology described by Malavolta et al. (1997).

#### 2.6.1 Gas exchange and photosynthetic pigments

An infrared gas analyzer (IRGA, model CIRAS-3, PP Systems) was used to determine the net photosynthetic rate (*A*) (µmol m^-^² s^-^¹), stomatal conductance (*gs*) (mol m^-^² s^-^¹), carbon concentration in the substomatal chamber (*Ci*) (µmol mol^-^¹), transpiration (*E*) (mmol m^-^² s^-^¹), carboxylation efficiency (*A/Ci*) and water use efficiency (*A/E*; WUE). For soybean, samples were taken from the central leaflet of the fully expanded third leaf and the intact trifoliate leaf from the apex of the main stem plant from 5 plants per plot. For maize and cotton, the third fully expanded leaf, counting from the apex to the base, was sampled from 5 plants per plot. All evaluations were performed in the morning, between 9 am and 11 am, with a constant ambient CO_2_ of 390 µmol mol^-^¹. For each crop, the readings were performed five days after the last application of MAP; thus, the readings were performed at R_3_+5 for soybean, R_1_+5 for maize and C_4_+5 for cotton.

To determine the leaf contents of the photosynthetic pigment’s chlorophyll *a*, chlorophyll *b*, total carotenoids and total chlorophylls, five discs with a diameter of 0.5 cm were cut from the last fully expanded leaf, between the edge and the central vein. The leaf samples were stored for 24 h in 2 mL of N,N-dimethylformamide (DMF) in glass vials wrapped in aluminum foil (Lichtenthaler, 1987). Pigment contents were quantified spectrophotometrically at wavelengths of 664, 647 and 480 nm for chlorophylls *a* and *b* and carotenoids, respectively (Wellburn, 1994).

#### 2.6.2 RuBisCO activity

Total RuBisCO activity was measured according to the method described by Reid et al. (1997). Frozen plant material (0.3 g) was ground with a mortar and pestle under liquid nitrogen and suspended in 1.5 mL of extraction buffer [58 mM potassium phosphate and 1 mM ethylenediaminetetraacetic acid (EDTA)]. The homogenized material was centrifuged at 14,000 rpm for 25 min at 4°C, and the supernatant was stored at 4°C (Reid et al., 1997; Sage et al., 2012).

The RuBisCO incubation buffer contained 100 mM bicine-NaOH pH 8.0, 25 mM potassium bicarbonate (KHCO_3_), 20 mM magnesium chloride (MgCl_2_), 3.5 mM ATP, 5 mM phosphocreatine, 0.25 mM NADH, 80 nkat glyceraldehyde-3-phosphate dehydrogenase, 80 nkat 3-phosphoglycerin phosphokinase, and 80 nkat creatine phosphokinase. Prior to initiating oxidation, 70 μL of the supernatant was incubated with 900 μL of the incubation buffer at 30°C for 5 min in the absence of ribulose-1,5-bisphosphate (RuBP) to allow carbamylation of RuBisCO. NADP oxidation was initiated by adding 30 µL of 16.66 mM RuBP directly to the cuvette. Readings were obtained on a spectrophotometer at a wavelength of 340 nm. RuBisCO activity was calculated from the difference in absorbance readings at 0 and 1 min (obtained without removing the cuvette from the spectrophotometer) and expressed in μmol min^–1^ mg protein^–1^ (Bossolani et al., 2021).

#### 2.6.3 Oxidative stress

H_2_O_2_ content was determined by referencing a calibration curve and expressed in µmol g^−1^ fresh weight (FW) (Alexieva et al., 2001). SOD activity (Giannopolitis and Ries, 1977) and expressed in units (U) mg^−1^ protein. CAT activity was assessed and expressed in µmol min^−1^ mg^−1^ protein (Azevedo et al., 1998). APX activity was expressed in nmol min^−1^ mg^−1^ protein (Gratão et al., 2008).

#### 2.6.4 Proline content

Proline content was determined according to Torello and Rice (1986). The absorbance at wavelengths of 647 and 664 nm was determined in a spectrophotometer, and the results were expressed per gram of FW (µmol g^−1^ FW) (Mauad et al., 2016).

#### 2.6.5 Metabolites

The same leave samples used in the nutritional analysis were used to analyze the contents of reducing sugars, total sugars, starch, and sucrose (Nelson, 1944).

### 2.7 Productivity parameters

For soybean, the final population, plant height, and numbers of branches, pods and grains per plant were determined from 10 plants in sequence in each plot at the R_8_ phenological stage. The 100-grain weight (13% moisture on a wet basis) and grain productivity were determined from a 4-m^2^ area in each plot and converted to kg ha^-1^ (13% moisture on a wet basis).

For maize, the final population, plant height, number of rows per ear, number of grains per row, 100-grain weight and productivity (13% moisture on a wet basis) were measured at physiological maturity by harvesting 10 ears per plot. The 100-grain weight was subsequently converted to bags per hectare.

The useful area of each cotton plot (2 m in 2 central rows) was harvested manually, and the final population, plant height, number of fruiting stems, and number of bolls per plant were measured. In addition, the boll mass and plume and seed productivity were determined and converted to kg ha^-1^. The seeds were separated from the plumes to analyze fiber quality (micronaire, length, resistance, and % short fiber) (Fonseca and Santana, 2002). The evaluations were carried out with the aid of a High-Volume Instrument (HVI).

### 2.8 Statistical analysis

The data were first analyzed for normality of errors (Shapiro and Wilk, 1965) and homoscedasticity of variances (Levene, 1960). Next, statistical analysis was performed using a double factorial design (treatments *vs*. growing seasons). The first factor was the application of soluble MAP, and the second factor was the growing season (2020/2021 or 2021/2022). When significant differences were detected by ANOVA (*p* ≤ 0.05), means were compared using Fischer’s protected t-test (LSD) at a 5% probability level. This analysis is summarized in the **Supplementary Material**. Since no significant effects of growing season or interactions between factors were observed, the averages of the two growing seasons are presented for each treatment.

## 3 Results

### 3.1 Soybean

**Figure 4:**
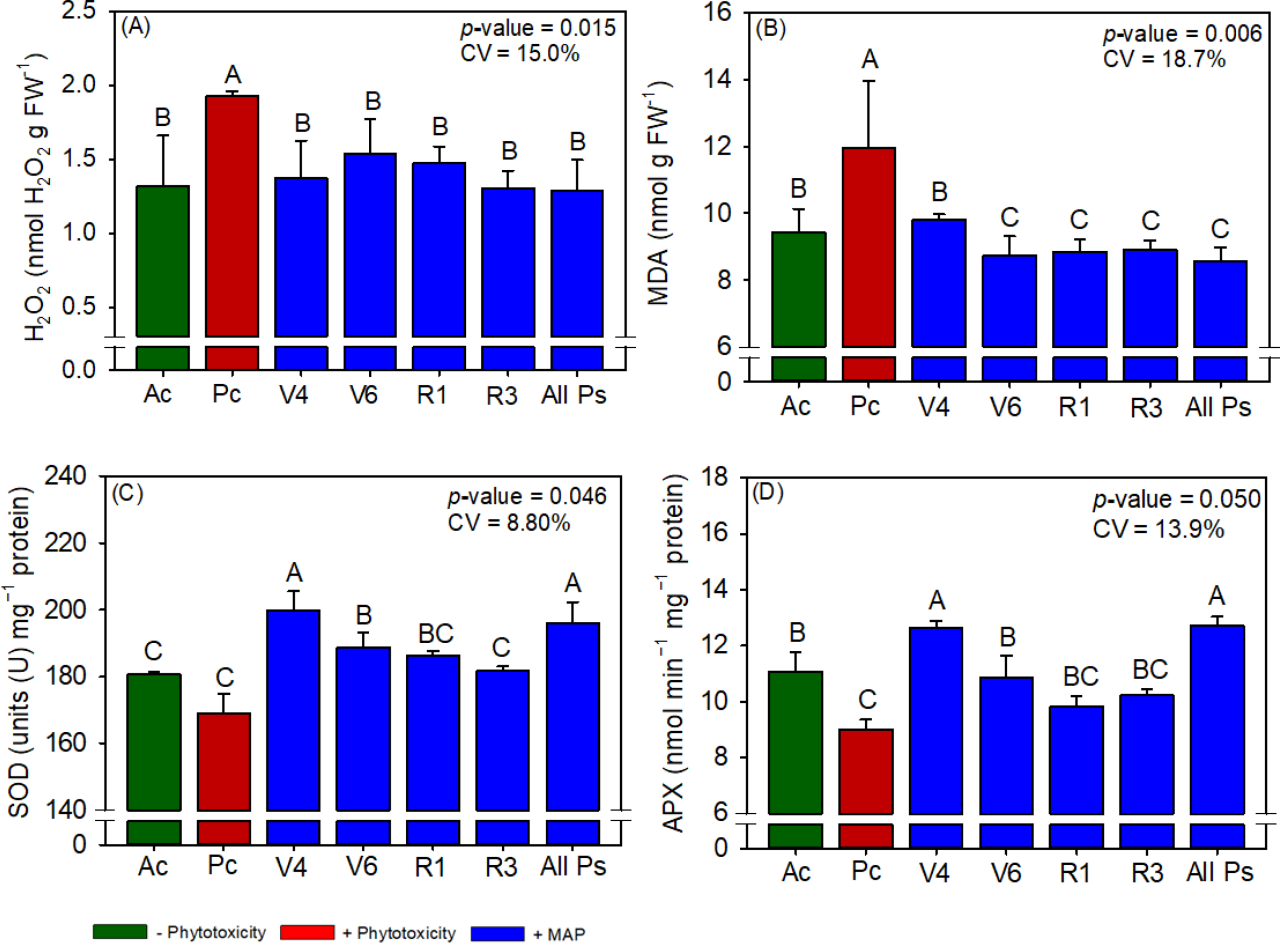
Response of various parameters – **(A)** H_2_O_2_, **(B)** MDA, **(C)** SOD and **(D)** APX activity’s — as a function of foliar soluble MAP application in soybean leaves. Bars for the same crop with different letters are significantly different by Fisher’s protected least significant difference (LSD) test at *p* ≤ 0.05. Growing seasons were considered random effects.

**Figure 5:**
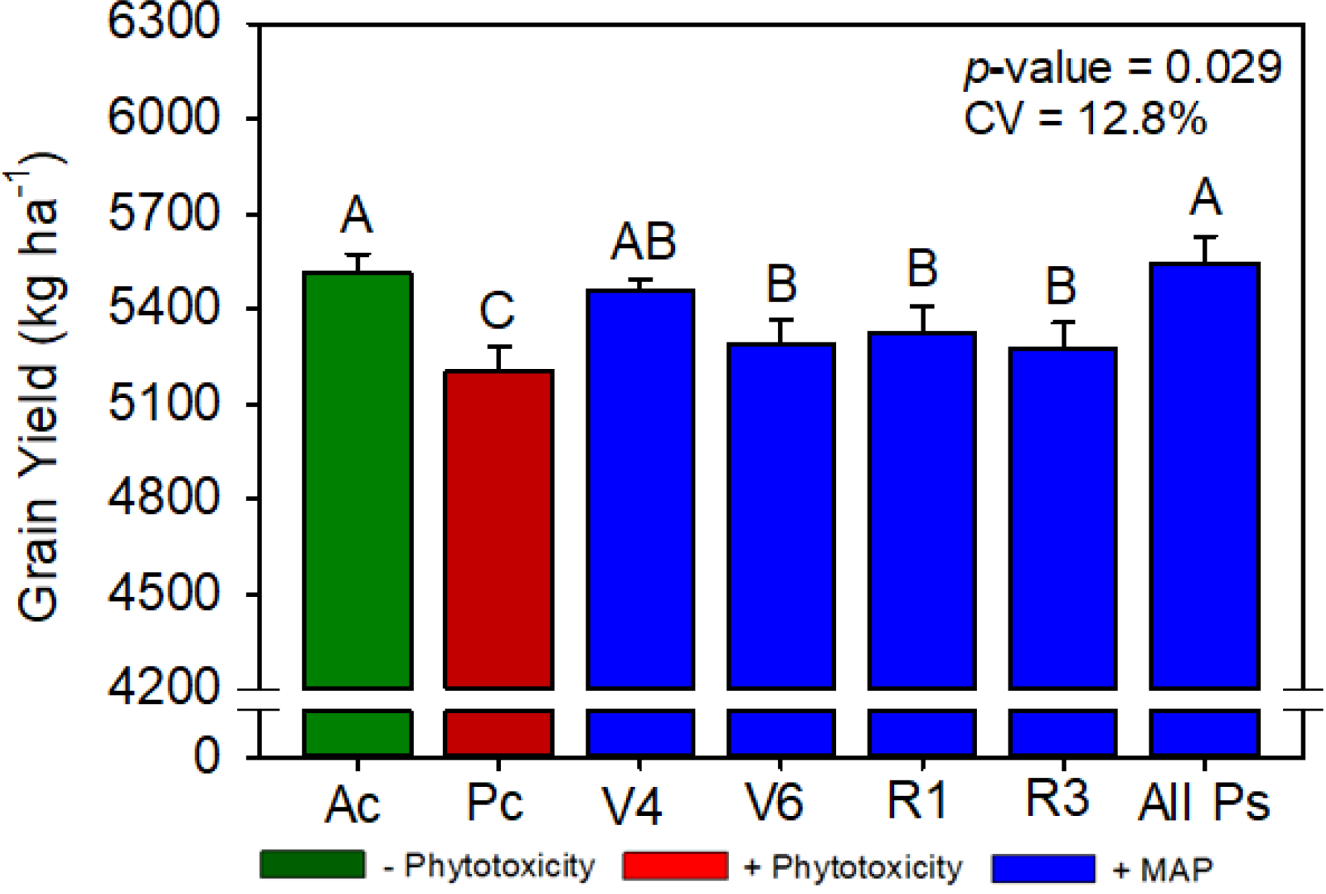
Soybean grain yield as a function of foliar soluble MAP application in soybean leaves. Bars with different letters are significantly different by Fisher’s protected least significant difference (LSD) test at *p* ≤ 0.05. Growing season was considered a random effect.

Soybean leaf P and N contents were not significantly different between the treatments (**Supplementary Table 1**). Compared with Ac, the induction of phytotoxicity (Pc) in soybean significantly reduced chlorophyll content, APX activity, and grain yield and significantly increased starch content, H_2_O_2_ content, and MDA content (**Figures 3**–**5**). The foliar application of soluble MAP eliminated these effects of phytotoxicity induction.

#### 3.1.1 Soybean chlorophyll and carotenoid content

The foliar application of soluble MAP had the greatest benefits for photosynthetic pigment content when it was performed at stages V_4_, V_6_, R_1_, and R_3_ (All Ps). Compared with Pc, All Ps increased chlorophyll *a* content by 13.2% (**Figure 3A**), chlorophyll *b* content by 15.2% (**Figure 3B**), and total chlorophyll content by 13.8% (**Figure 3C**). The contents of these pigments in All Ps were not significantly different from those in Ac. Neither phytotoxicity induction nor foliar MAP application significantly affected carotenoid content (**Supplementary Table 2**).

#### 3.1.2 Soybean metabolite content

Regardless of timing, the application of soluble MAP reduced starch production by approximately 23% compared with Pc (**Figure 3D**). The contents of reducing and total sugars did not differ significantly between the treatments (**Supplementary Table 3**).

#### 3.1.3 Soybean RuBisCO activity and gas exchange

All gas exchange parameters and RuBisCO activity were not significantly different between the treatments (**Supplementary Tables 3**, **4**).

#### 3.1.4 Soybean antioxidant enzyme activity and oxidative stress

Conversely, soluble MAP application at this growth stage reduced H_2_O_2_ content by 33.7% (**Figure 4A**) and MDA content by 28.3% (**Figure 4B**) compared to Pc. CAT activity and proline content were not significantly different between the treatments (**Supplementary Table 5**). Although phytotoxicity induction did not significantly reduce SOD activity (**Figure 4C**), the foliar application of soluble MAP in stage V_4_ increased SOD activity by 10.7% compared with Pc. Moreover, MAP application at this growth stage restored APX activity to levels surpassing those in Ac, with an increase of 40.6% compared with Pc (**Figure 4D**).

#### 3.1.5 Soybean productivity parameters

Compared with Pc, All Ps increased the soybean grain yield by 6.0% (**Figure 5**). Regardless of timing, foliar MAP application rescued the decrease in plant height caused by phytotoxicity induction (**Supplementary Table 6**). Plant population, 100-grain weight, and number of branches were not significantly different between the treatments (**Supplementary Tables 6**, **7**).

### 3.2 Maize

**Figure 6:**
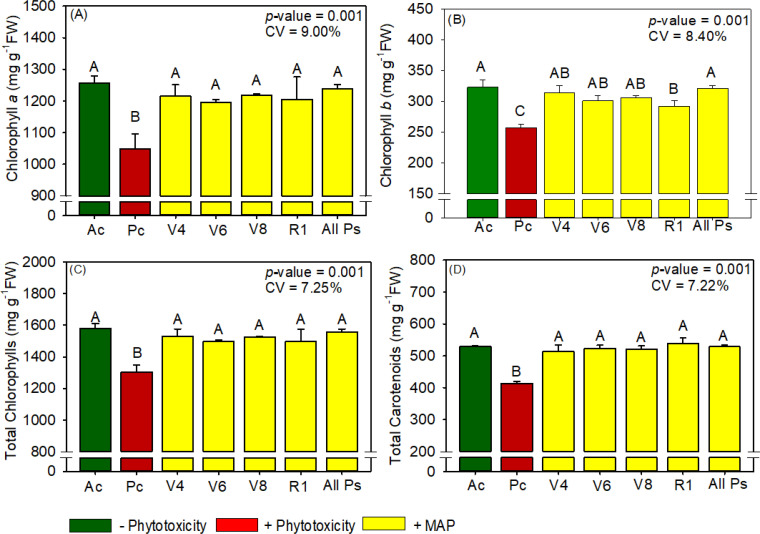
Response of various parameters — **(A)** chlorophyll *a*, **(B)** chlorophyll *b*, **(C)** total chlorophyl and **(D)** total carotenoids — as a function of foliar soluble MAP application in maize leaves. Bars with different letters are significantly different by Fisher’s protected least significant difference (LSD) test at *p* ≤ 0.05. Growing season was considered a random effect.

**Figure 7:**
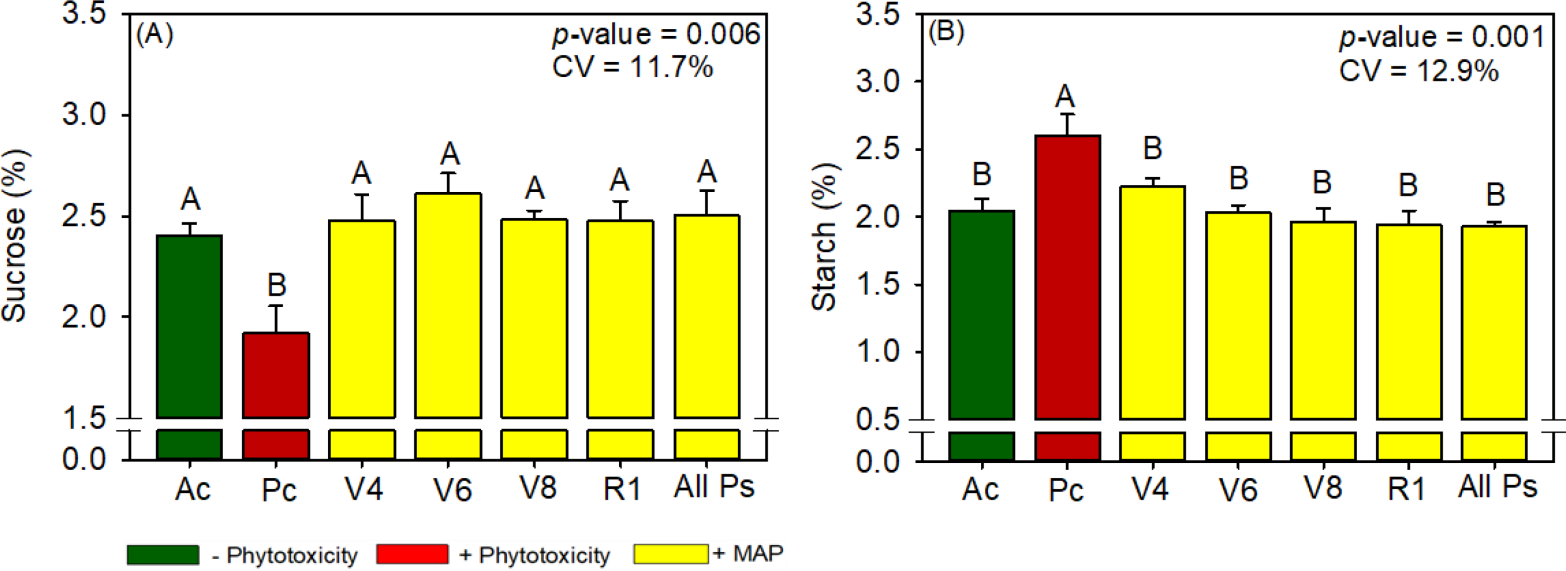
Response of various parameters — **(A)** sucrose and **(B)** starch — as a function of foliar soluble MAP application in maize leaves. Bars with different letters are significantly different by Fisher’s protected least significant difference (LSD) test at *p* ≤ 0.05. Growing season was considered a random effect.

**Figure 8:**
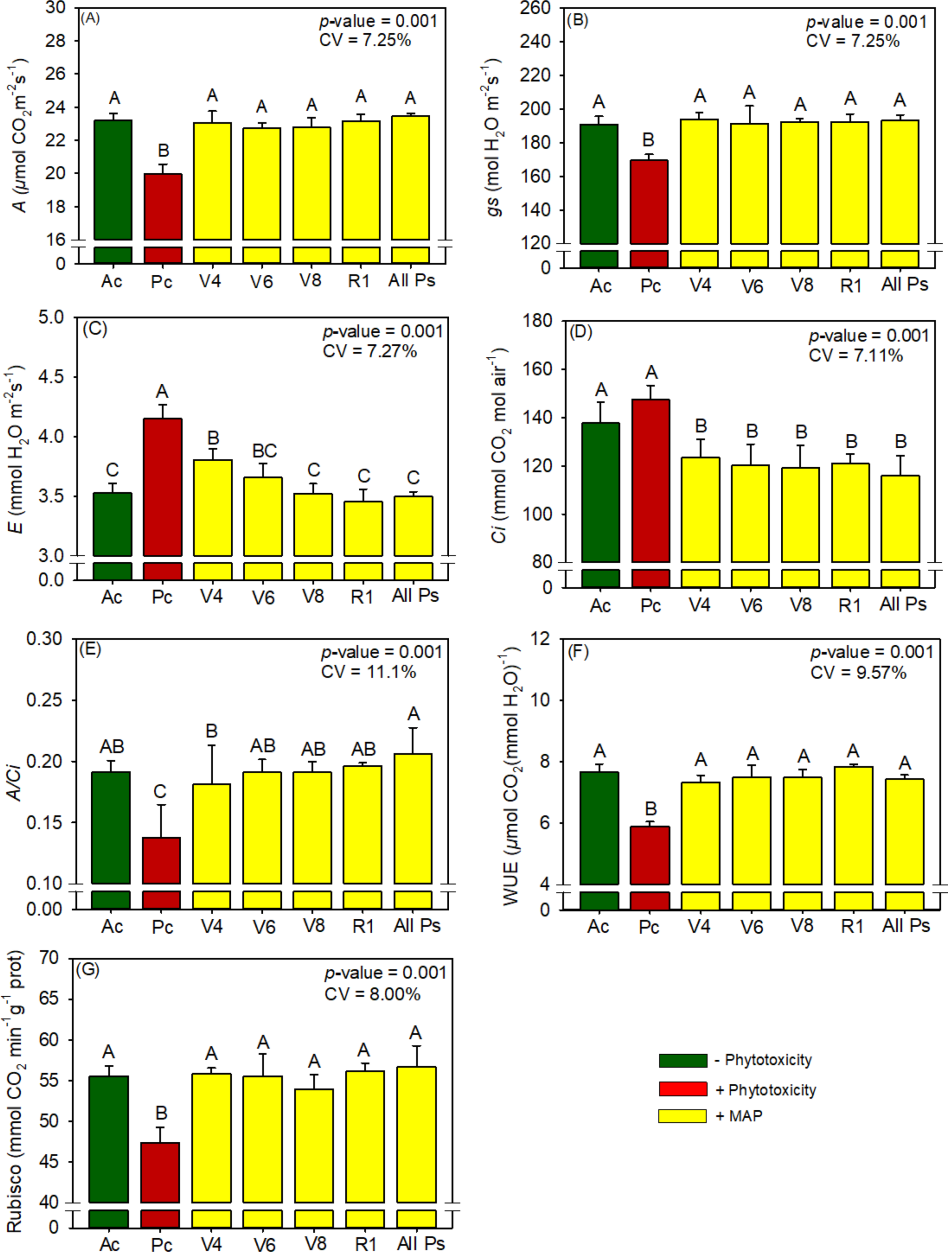
Response of various parameters — **(A)** *A*, **(B)** *gs*, **(C)** *E*, **(D)** *Ci*, **(E)** *A/Ci*, **(F)** *WUE*, and **(G)** RuBisCO activity — as a function of foliar soluble MAP application in maize leaves. Bars with different letters are significantly different by Fisher’s protected least significant difference (LSD) test at *p* ≤ 0.05. Growing season was considered a random effect.

**Figure 9:**
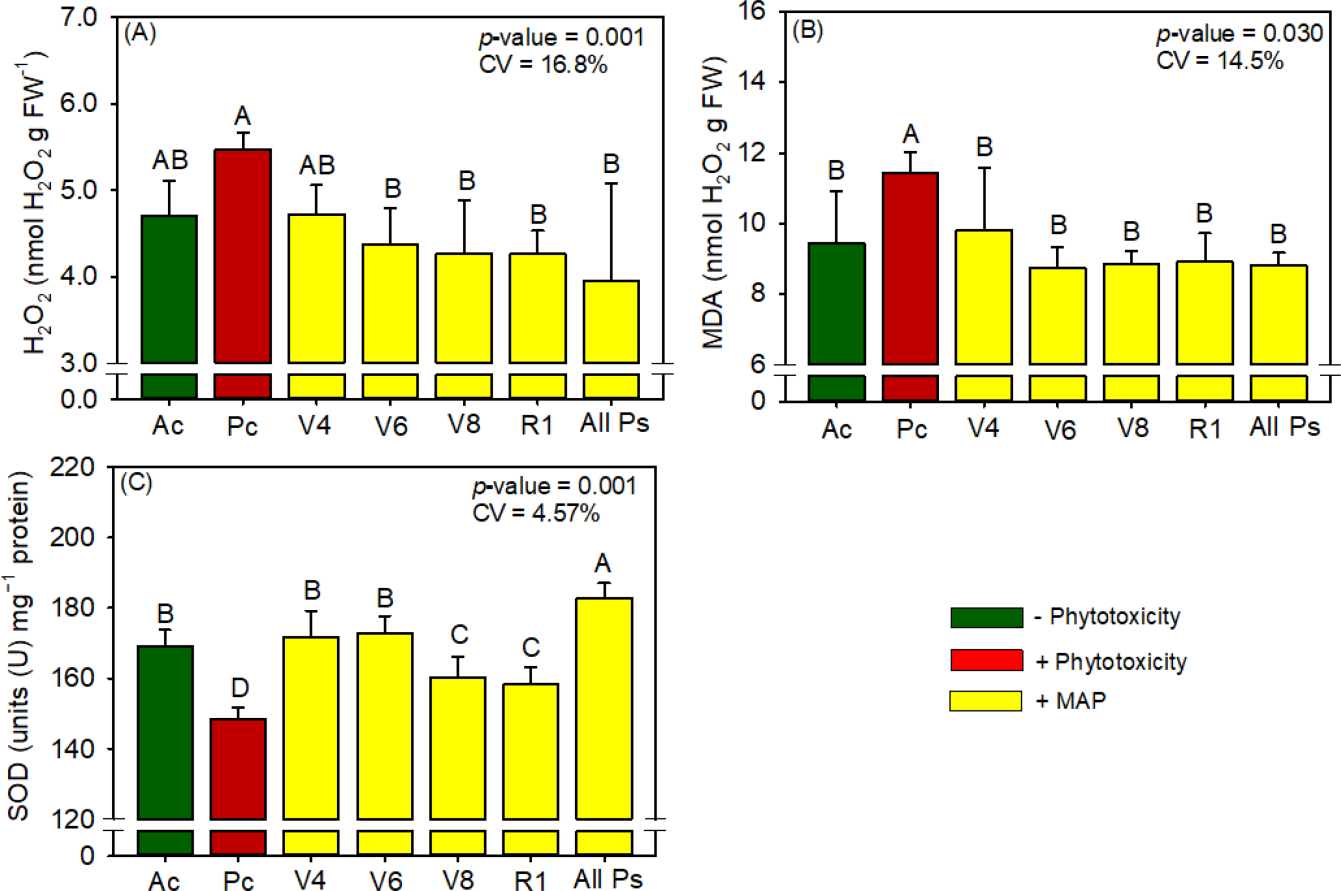
Response of various parameters — **(A)** H_2_O_2_, **(B)** MDA and **(C)** SOD activity’s — as a function of foliar soluble MAP application in maize leaves. Bars for the same crop with different letters are significantly different by Fisher’s protected least significant difference (LSD) test at *p* ≤ 0.05. Growing seasons were considered random effects.

**Figure 10:**
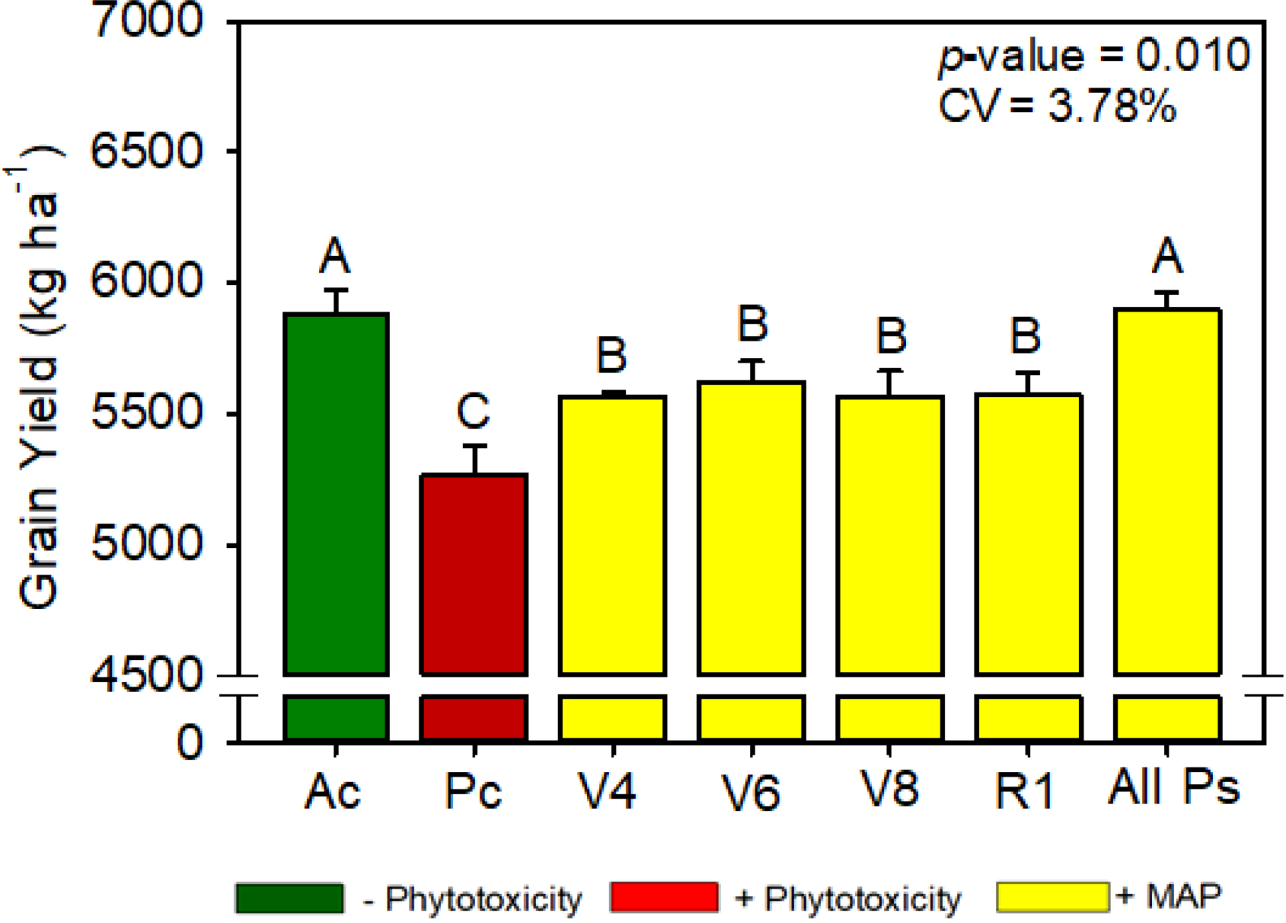
Maize grain yield as a function of foliar soluble MAP application in maize leaves. Bars with different letters are significantly different by Fisher’s protected least significant difference (LSD) test at *p* ≤ 0.05. Growing season was considered a random effect.

Maize leaf P and N contents were not significantly different between the treatments (**Supplementary Table 8**). Compared with Ac, Pc significantly reduced chlorophyll content, carotenoid content, sucrose content, RuBisCO activity, *A*, *gs*, *A/Ci*, WUE, SOD activity, 100-grain weight, and grain yield of maize and significantly increased starch content, *E*, *Ci*, and MDA content (**Figures 6**–**10**). Similar to the effects observed in soybean, the foliar application of soluble MAP reversed these effects of phytotoxicity induction.

#### 3.2.1 Maize chlorophyll and carotenoid content

Compared with Pc, All Ps increased chlorophyll *a* content by 15.1% (**Figure 6A**), chlorophyll *b* content by 24.8% (**Figure 6B**), total chlorophyll content by 20.5% (**Figure 6C**), and carotenoid content by 26.9% (**Figure 6D**). The contents of these photosynthetic pigments were not significantly different between All Ps and Ac (**Supplementary Table 9**).

#### 3.2.2 Maize metabolite content

Regardless of timing, the foliar application of soluble MAP significantly increased sucrose content (**Figure 7A**) by approximately 29% compared with Pc. The enhancement of sucrose content was accompanied by a significant reduction in starch production of approximately 25% (**Figure 7B**; **Supplementary Table 11**). These changes reflect a shift in carbohydrate allocation, with an increase in soluble sugars and a corresponding decrease in starch accumulation. The sucrose and starch contents in the MAP treatments were not significantly different from those in Ac. Reducing sugar and total sugar contents were not significantly different between the treatments (**Supplementary Table 10**).

#### 3.2.3 Maize RuBisCO activity and gas exchange

The foliar application of MAP also had significant benefits for gas exchange in maize (**Supplementary Table 12**): compared with Pc, *A* increased by 15.8% (**Figure 8A**), *gs* increased by 14.0% (**Figure 8B**), *E* decreased by 15.9% (**Figure 8C**), and *Ci* decreased by 21.4% (**Figure 8D**). Overall, MAP application increased *A/Ci* by 45% (**Figure 8E**) and by 33.4% (**Figure 8F**). MAP application increased RuBisCO activity by an average of 18.5% compared with Pc (**Figure 8G**; **Supplementary Table 11**), regardless of the timing of application.

#### 3.2.4 Maize antioxidant enzyme activity and oxidative stress

These increases in activity were accompanied by decreases in H_2_O_2_ (**Figure 9A**) and MDA (**Figure 9B**) contents of 27.8% and 22.6%, respectively. APX and CAT activities and proline content did not differ significantly between the treatments (**Supplementary Table 13**). Compared with Pc, All Ps significantly increased the activity of the antioxidant enzyme SOD (**Figure 9C**) by 23.3%.

#### 3.2.5 Maize productivity parameters

The application of soluble MAP significantly increased productivity compared with Pc, with a 12% boost in grain yield (**Figure 10**). The final plant population, plant height, number of rows per ear, number of grains per row and 100-grains weight were not significantly different between the treatments (**Supplementary Tables 14**, **15**).

### 3.3 Cotton

**Figure 11:**
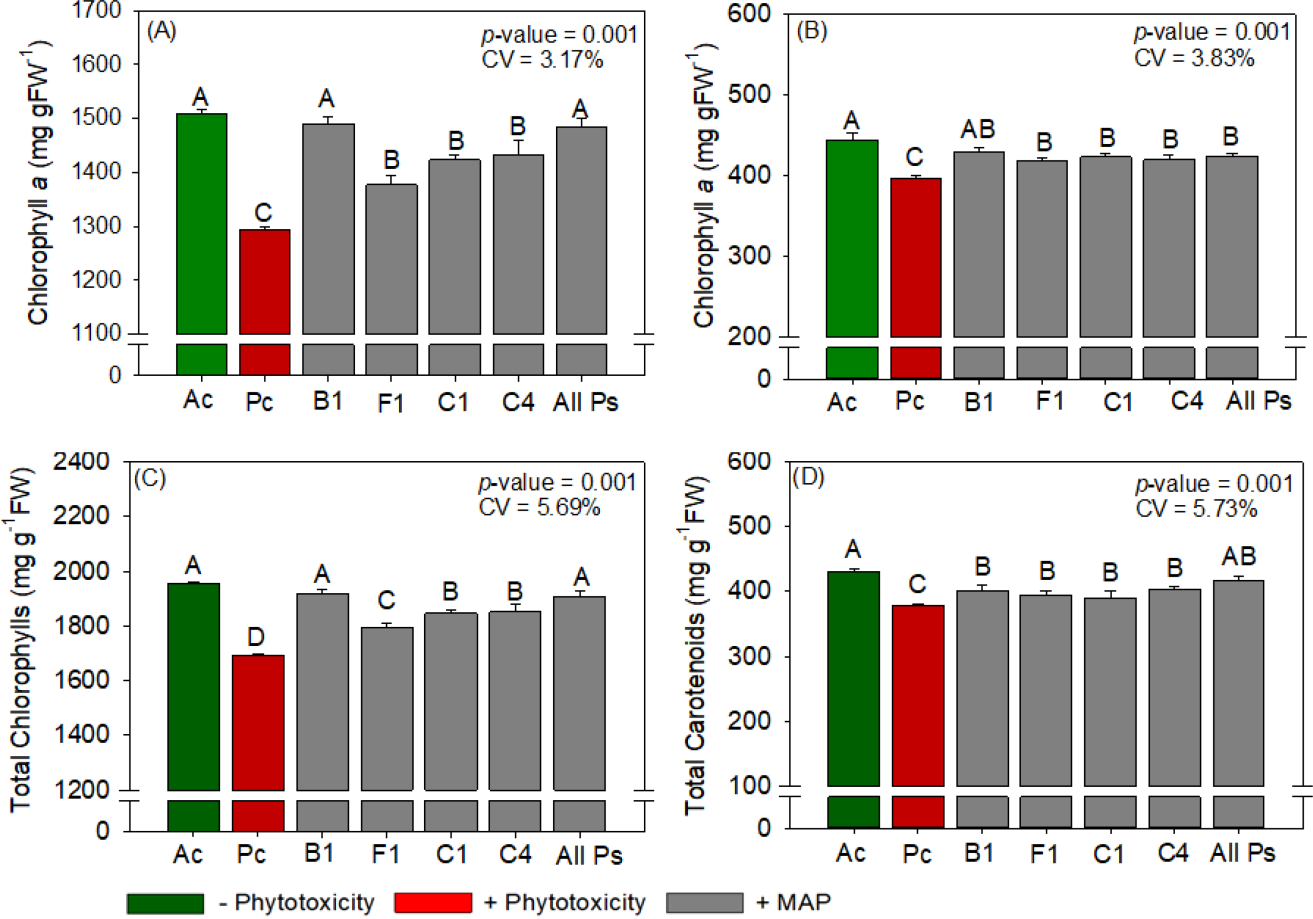
Response of various parameters — **(A)** chlorophyll *a*, **(B)** chlorophyll *b*, **(C)** total chlorophyl and **(D)** total carotenoids — as a function of foliar soluble MAP application in cotton leaves. Bars with different letters are significantly different by Fisher’s protected least significant difference (LSD) test at *p* ≤ 0.05. Growing season was considered a random effect.

**Figure 12:**
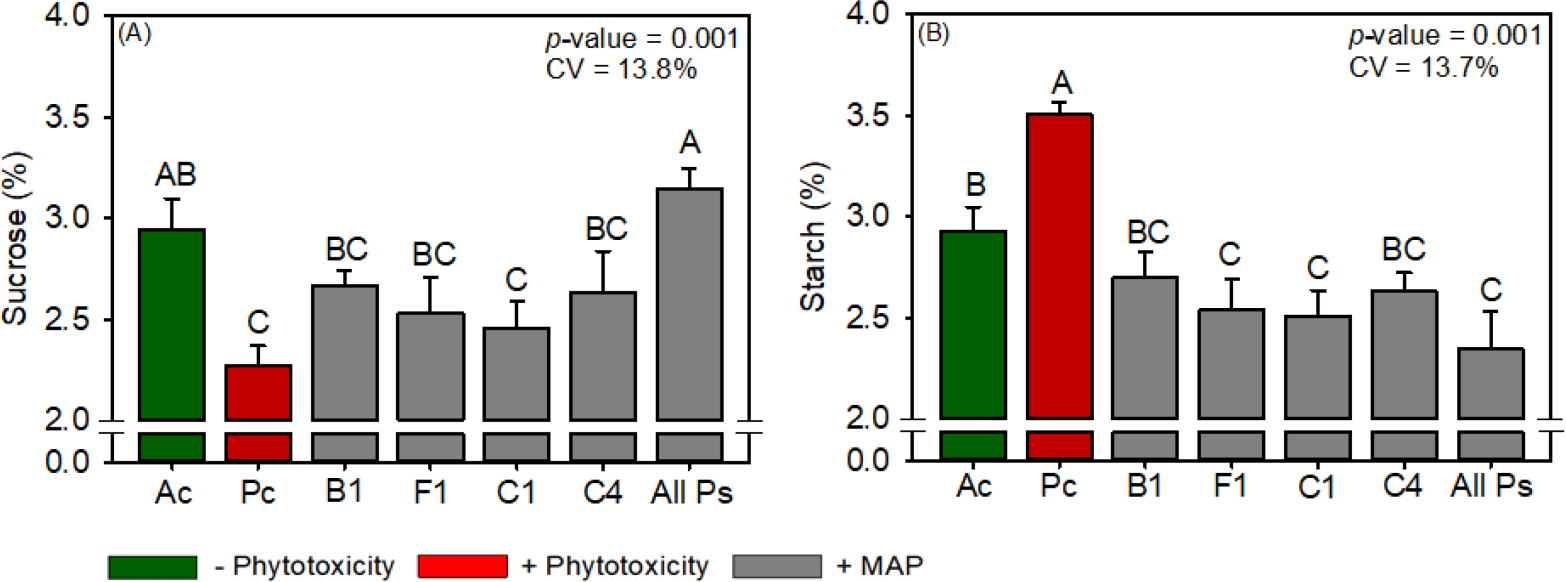
Response of various parameters — **(A)** sucrose and **(B)** starch — as a function of foliar soluble MAP application in cotton leaves. Bars with different letters are significantly different by Fisher’s protected least significant difference (LSD) test at *p* ≤ 0.05. Growing season was considered a random effect.

**Figure 13:**
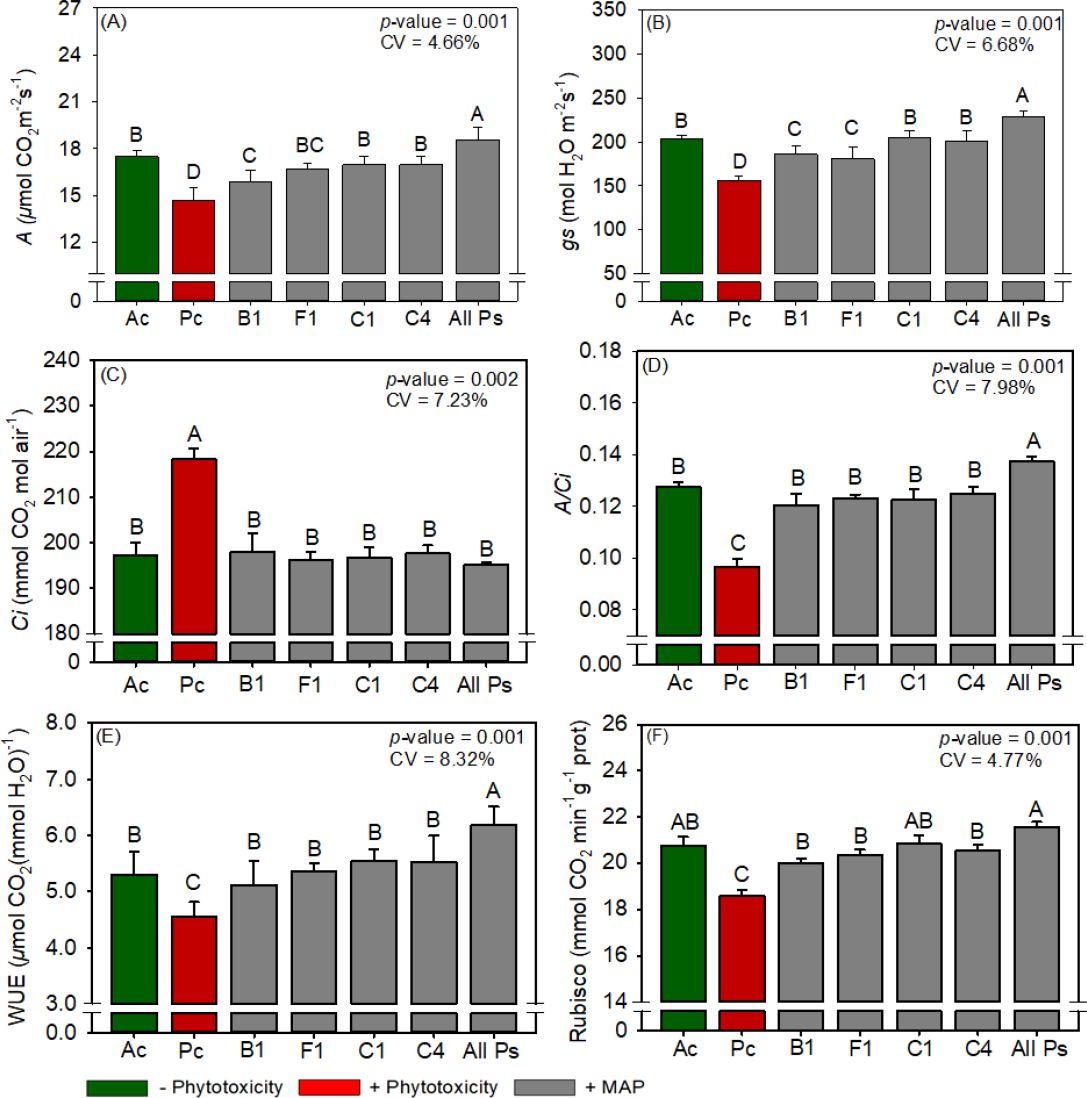
Response of various parameters — **(A)** *A*, **(B)** *gs*, **(C)** *Ci*, **(D)** *A/Ci*, **(E)** *WUE* and **(F)** RuBisCO activity — as a function of foliar soluble MAP application in cotton leaves. Bars with different letters are significantly different by Fisher’s protected least significant difference (LSD) test at *p* ≤ 0.05. Growing season was considered a random effect.

**Figure 14:**
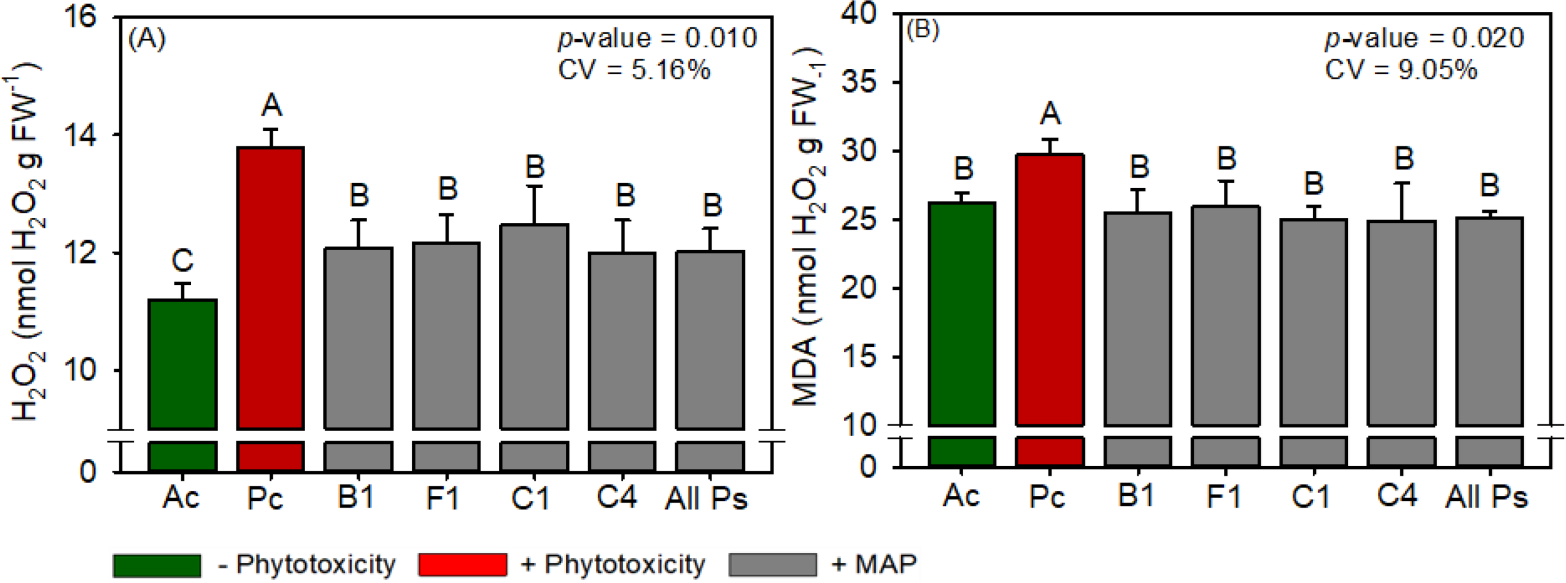
Response of various parameters — **(A)** H_2_O_2_ and **(B)** MDA — as a function of foliar soluble MAP application in cotton leaves. Bars for the same crop with different letters are significantly different by Fisher’s protected least significant difference (LSD) test at *p* ≤ 0.05. Growing seasons were considered random effects.

**Figure 15:**
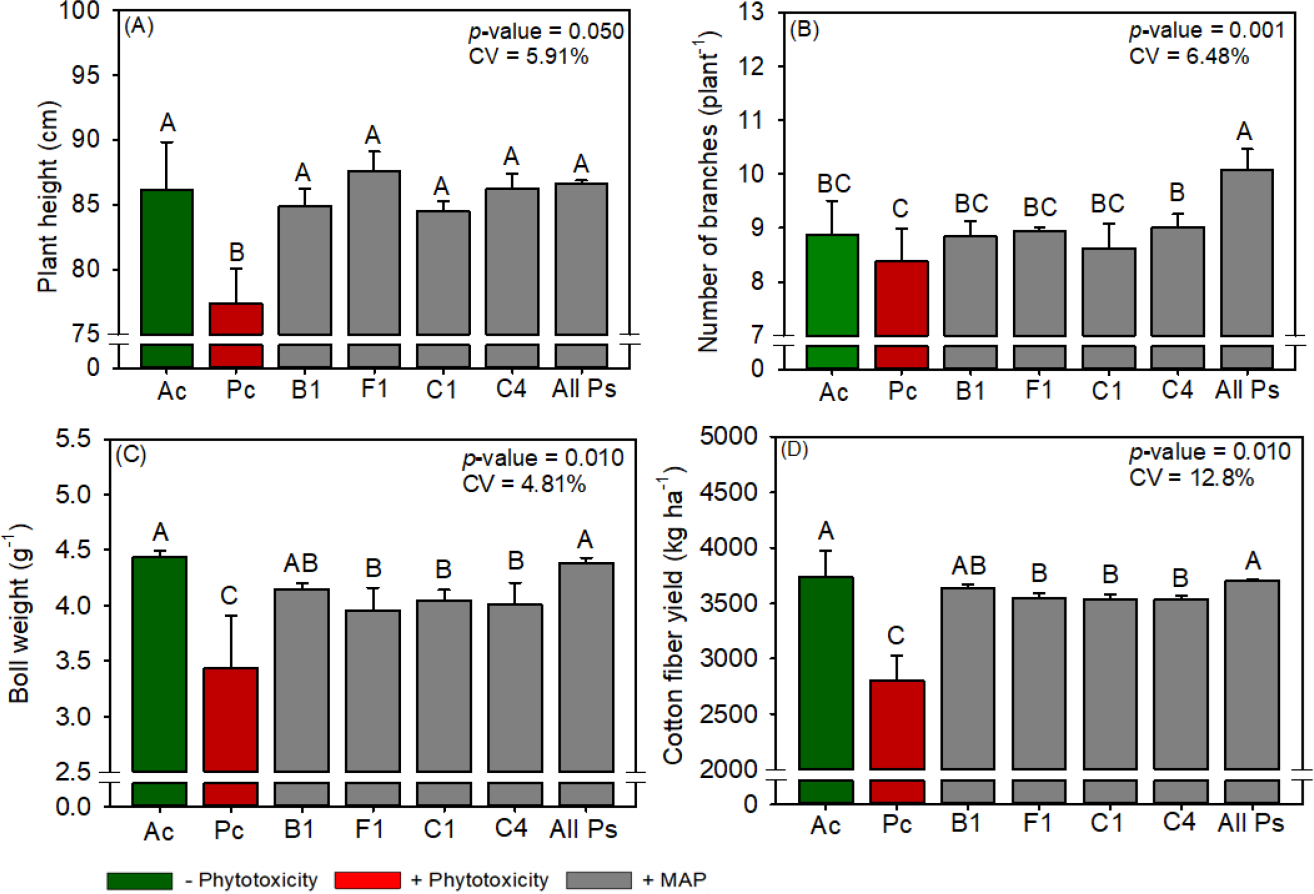
Response of various parameters — **(A)** plant height, **(B)** number of branches, **(C)** boll weight and **(D)** cotton fiber yield —as a function of foliar soluble MAP application in cotton leaves. Bars with different letters are significantly different by Fisher’s protected least significant difference (LSD) test at *p* ≤ 0.05. Growing season was considered a random effect.

The leaf P and N contents of cotton were not significantly different between the treatments (**Supplementary Table 16**). Compared with Ac, Pc significantly reduced chlorophyll content, carotenoid content, sucrose content, RuBisCO activity, *A*, *gs*, *A/Ci*, WUE, plant height, boll weight, and cotton fiber yield but significantly increased starch content, *Ci*, H_2_O_2_ content, and MDA content (**Figures 11**–**15**). MAP application generally eliminated these effects of phytotoxicity induction, consistent with the results for soybean and maize.

#### 3.3.1 Cotton chlorophyll and carotenoid content

Foliar application of soluble MAP at stage B_1_ had the greatest positive effects on photosynthetic pigment content; in general, pigment levels in treatment B_1_ were not significantly different from those in Ac (**Figure 11**; **Supplementary Table 17**). Compared with Pc, application at B_1_ increased chlorophyll *a* content by 14.7% (**Figure 11A**), chlorophyll *b* content by 8.5% (**Figure 11B**), total chlorophyll content by 12.8% (**Figure 11C**), and total carotenoid content by 10% (**Figure 11D**).

#### 3.3.2 Cotton metabolite content

Compared to Pc, All Ps increased sucrose content by 38.2% (**Figure 12A**; **Supplementary Table 18**) and reduced starch content by 33.1% (**Figure 12B**; **Supplementary Table 19**). Reducing sugar and total sugar contents were not significantly different between the treatments (**Supplementary Table 18**).

#### 3.3.3 Cotton RuBisCO activity and gas exchange

The foliar supplementation with soluble MAP increased *A* by 26.3% (**Figure 13A**) and *gs* by 45.7% (**Figure 13B**), reduced *Ci* by up to 10.6% (**Figure 13C**), and improved WUE (**Figure 13E**) and *A/Ci* (**Figure 13D**) by 35.6% and 42.7%, respectively. *E* did not differ significantly between the treatments (**Supplementary Table 20**). All Ps increased RuBisCO activity by 15.9% compared with Pc (**Figure 13F**; **Supplementary Table 19**).

#### 3.3.4 Cotton antioxidant enzyme activity and oxidative stress

Compared with Pc, All Ps decreased the leaf contents of H_2_O_2_ (**Figure 14A**) and MDA (**Figure 14B**) by 13% and 14.3%, respectively, and the contents of H_2_O_2_ and MDA in All Ps were not significantly different from those in Ac. The activities of antioxidant enzymes and proline content did not differ significantly between the treatments (**Supplementary Table 21**).

#### 3.3.5 Cotton productivity parameters and fiber quality

All Ps increased cotton plant height by 6.34% (**Figure 15A**), the number of branches per plant by 14.9% (**Figure 15B**), boll weight by 19% (**Figure 15C**), and fiber yield by 9.7% (**Figure 15D**) compared to Pc. Conversely, foliar MAP application decreased the short fiber index (SFI) by an average of 8.2% compared to Pc. The plant population and number of bolls per plant did not differ between the treatments (**Supplementary Tables 22**, **23**).

## 4 Discussions

Crop productivity and grain and fiber quality are the outcomes of primary plant processes that regulate the rates of absorption, assimilation and distribution of nutrients and biomass (Rogeri et al., 2017; Saleem et al., 2023). Some of the factors that influence these processes are controllable, such as phytotechnical management and fertilization (Crusciol et al., 2022). Traditional soil fertilization serves a clear and specific purpose: to supplement the quantity and quality of nutrients provided by the soil for plant growth. Similarly, foliar fertilization must have well-defined objectives, guided by technical and/or economic considerations, such as mitigating oxidative stress (Fernández and Brown, 2013).

Oxidative stress reduces the photosynthetic rate, leading to an increase in *Ci* (Moretti et al., 2021). The low availability of CO_2_ caused by stomatal closure may reduce the ability of photosystem II to maintain an adequate balance between electron transport, carbonmetabolism, and ATP and NADPH consumption (Hermans et al., 2004). Elevated *Ci* blocks electron transport and interrupts ATP and NADPH production, leaving the plant unable to assimilate available CO_2_ for conversion into energy products (Bari et al., 2020).

In the present study, the induction of oxidative stress by herbicide application displaced the entire stocks of N and P in the leaves of the crops toward the recovery of the affected photosynthetic processes. P participates in chlorophyll production in the form of ATP, and N is found in the pyrrolic rings of chlorophylls, in which a central magnesium atom is linked to four N atoms (Fiedor et al., 2008). Failure to supplement P and N after phytotoxicity induction can negatively affect nucleic acid synthesis and the cell membrane and directly reduce chlorophyll content (Ahmad et al., 2021; Hand et al., 2021). Chlorophyll is responsible for capturing light energy and initiating photosynthetic activity, carbon metabolism, and antioxidant enzyme activity (Khan et al., 2023; Zhou et al., 2023). The herbicide carfentrazone-ethyl inhibits PPOX, an enzyme in the pathway for the synthesis of chlorophyll *a*. Inhibiting PPOX not only reduces chlorophyll levels but also increases ROS formation due to the reaction of protoporphyrinogen IX accumulated in the cytoplasm with light (Sánchez-Moreiras et al., 2020). Our results demonstrate that foliar fertilization with MAP can restore chlorophyll production after oxidative stress.

Foliar fertilization with MAP increased gas exchange parameters, which are linked to carbon fixation activity, and the production of sucrose. Sucrose is the main sugar for transport in many plants, and its production is favored by the increased availability of Pi (inorganic phosphate) provided via foliar MAP application (Pelá et al., 2019). Greater Pi availability increases energy capacity and facilitates the movement of triose molecules from the cytoplasm to the cytosol, initiating sucrose synthesis. Conversely, the absence of P supplementation favors starch production in chloroplasts (Maheshwari et al., 2021). These effects of foliar MAP fertilization are part of a chain linked to the recovery of chlorophyll levels.

Throughout the photosynthetic process, CO_2_ plays the role of substrate. It diffuses into plant cells through the stomata; thus, plants with higher stomatal conductance have a greater capacity to balance CO_2_ uptake with water loss through transpiration (Taiz et al., 2017). Our results were obtained during a period following P and N supplementation. To obtain a better understanding of the effects of foliar fertilization with N and P on gas exchange and photosynthetic processes, studies throughout the entire crop cycle are needed.

Foliar MAP fertilization of soybean, maize, and cotton to attenuate phytotoxicity caused by the herbicide carfentrazone-ethyl increased *A*, *gs*, and WUE and consequently reduced *E*. In addition, *Ci* decreased, and *A/Ci* increased. These effects reflect an increase in the rate of carbon assimilation by RuBisCO, an enzyme present in all photosynthetic organisms (Kubien et al., 2008). Foliar fertilization with MAP increased RuBisCO activity by providing N and P, which are components of RuBisCO and chlorophyll (Xu et al., 2012; Guo et al., 2016). Increasing the production of photosynthetic components promotes the accumulation of organic compounds, which act in cellular osmotic adjustment and contribute to photosynthetic efficiency (Xu et al., 2012). Collectively, our effects illustrate the benefits of foliar MAP application for improving photosynthetic efficiency and optimizing water use and gas exchange processes in crops.

The herbicide carfentrazone-ethyl does not directly interrupt photosynthetic processes. However, direct contact between the plant and the herbicide interrupts chlorophyll production and increases the accumulation of compounds that lead to the formation of singlet oxygen (Holmes et al., 2022; Li et al., 2022). The resulting lipid peroxidation and membrane disruption negatively affect photosynthesis, respiration, and electron transport (Sahu et al., 2023). Nutrients play crucial roles in photosynthetic processes; for example, P is involved in electron transport, and N is involved in chloroplast formation and protein synthesis, activates enzymes, and contributes to plant biomass production (Moreira et al., 2014, 2018; Rodrigues et al., 2021b). Thus, foliar fertilization with these nutrients under conditions of oxidative stress provides a resource for the recovery of plants affected by phytotoxicity (Hudina and Stampar, 2001; Zanao et al., 2020). Supplementation of plants with N under stress conditions increases nitrate-N absorption, nitrate reductase activity, and antioxidant defense mechanisms, reducing pigment photooxidation in chloroplasts and consequently in leaves (Kang et al., 2023). Oxidative stress may reduce the P content of the plant, limiting the growth of its root system (Ahmad et al., 2018). Foliar supplementation with P restores root growth and increases water and nutrient absorption, strengthening the plant’s defense system. Additionally, P supplementation increases nitrate reductase activity, leading to greater nitrate assimilation under stress conditions (Ahmad et al., 2021; Viveiros et al., 2024).

In the present study, phytotoxicity induction reduced the activity of antioxidant enzymes in all three crops, and foliar supplementation with P and N restored high levels of enzyme activity. To assess the effects of phytotoxicity induction on lipid peroxidation, the leaf concentrations of MDA and H_2_O_2_ were evaluated. MDA is commonly used as an indicator of oxidative stress and is formed from the oxidation of polyunsaturated fatty acids (Rodrigues et al., 2021b; Viveiros et al., 2024). H_2_O_2_ is derived from the reduction of O_2_^-^ by SOD and is neutralized in two steps by CAT (Gill and Tuteja, 2010). Phytotoxicity induction increased the leaf contents of both MDA and H_2_O_2_, and these increases were reversed by foliar MAP application, consistent with the changes in SOD activity (Niu et al., 2021). SOD is the first line of defense against oxidative stress, and the increases in SOD activity in the treatments containing foliar MAP indicate that MAP application improved the plant’s ability to combat ROS (Almeselmani et al., 2006; Gong et al., 2020). These results highlight the positive impact of MAP application on boosting antioxidative enzymatic activity and mitigating oxidative stress.

Proline is a nitrogenous compound that contributes to the recovery of plant growth and combatting phytotoxicity (Torello and Rice, 1986; Rodrigues et al., 2021a). Surprisingly, herbicide application without foliar MAP fertilization did not significantly alter proline concentrations compared with the treatments with MAP application, in contrast to the effects of phytotoxicity induction on other indicators of oxidative stress. In summary, foliar MAP fertilization provides P and N in direct contact with the leaf, which improves photosynthetic efficiency, reduces ROS formation, and mitigates the effects of oxidative stress on plants (Fernández and Brown, 2013; Rodrigues et al., 2021b).

## 5 Conclusions

This study evaluated the ability of foliar fertilization with soluble MAP (containing N and P) to mitigate oxidative stress induced by the herbicide carfentrazone-ethyl in soybean, maize, and cotton. Foliar supplementation with MAP alleviated symptoms of phytotoxicity, regardless of the timing of MAP application. However, yields were highest when soluble MAP was applied in a total of four phenological stages. Phosphorus and N enhance plant defense and cellular recovery under stress by supporting energy transfer, protein synthesis, and antioxidant activation, ultimately improving resilience and productivity.

## Data Availability

The original contributions presented in the study are included in the article/**Supplementary Material**. Further inquiries can be directed to the corresponding author.
