# Supplementary material for: Can foliar application of soluble monoammonium phosphate effectively alleviate herbicide-induced oxidative stress in key crops?
Source: Front Plant Sci. 2025 Feb 28;16:1504244. doi: 10.3389/fpls.2025.1504244 (PMC11907198; doi:10.3389/fpls.2025.1504244)
Supplement: Supplementary file 1 [file Table1.docx]

SUPPLEMENTARY MATERIAL

**CAN FOLIAR APPLICATION OF SOLUBLE MONOAMMONIUM PHOSPHATE EFFECTIVELY ALLEVIATE HERBICIDE-INDUCED OXIDATIVE STRESS IN KEY CROPS?**

***SOYBEAN***

**TABLE 1.** Soybean leaf P and N contents as a function of the foliar application of MAP at different phenological stages, Botucatu - SP. Values in the same column with different letters are significantly different by Fisher's protected least significant difference (LSD) test at *p* ≤ 0.05. Growing season was considered a random effect.

| **Treatment Application (TA)** | **Foliar Phosphorus** | **Foliar Nitrogen** |
| --- | --- | --- |
|  | g kg^−1^ | |
| Absolute control (Ac) | 3.50 a | 30.7 a |
| Phytotoxicity control (Pc) ^a^ | 3.37 a | 30.9 a |
| V_4_ ^b^ | 3.40 a | 30.9 a |
| V_6_ ^b^ | 3.35 a | 29.5 a |
| R_1_ ^b^ | 3.35 a | 30.2 a |
| R_3_ ^b^ | 3.35 a | 29.1 a |
| All phenological stages (All Ps) ^b^ | 3.50 a | 31.0 a |
| **Cropping Season (CS)** |  |  |
| 2020–2021 | 2.96 a | 32.2 a |
| 2021–2022 | 3.83 a | 28.5 a |
| **ANOVA (*F probability*)** ^c^ |  |  |
| TA | 1.000 | 0.986 |
| CS | 0.230 | 0.180 |
| TA *x* CS | 0.770 | 0.524 |
| C.V. (%) | 20.7 | 18.4 |

^a^ Induction of moderate phytotoxicity by the herbicide carfentrazone-ethyl.

^b^ Application of soluble monoammonium phosphate (MAP) after induction of phytotoxicity at phenological stage V_3_.

^c^ There were no significant interactions between treatment applications or between cropping seasons.

**TABLE 2.** Soybean leaf contents of chlorophyll *a*, chlorophyll *b*, total chlorophyll and total carotenoids as a function of the foliar application of MAP at different phenological stages, Botucatu - SP. Values in the same column with different letters are significantly different by Fisher's protected least significant difference (LSD) test at *p* ≤ 0.05. Growing season was considered a random effect.

| **Treatment Application (TA)** | **Chlorophyll *a*** | **Chlorophyll *b*** | **Total Chlorophyll** | **Total Carotenoids** |
| --- | --- | --- | --- | --- |
|  | mg g^−1^FW | | | |
| Absolute control (Ac) | 1176 a | 531 a | 1707 a | 532 a |
| Phytotoxicity control (Pc) ^a^ | 1021 c | 447 c | 1468 c | 465 a |
| V_4_ ^b^ | 1108 b | 462 b | 1570 b | 491 a |
| V_6_ ^b^ | 1079 b | 433 c | 1512 bc | 484 a |
| R_1_ ^b^ | 1120 b | 482 b | 1593 b | 495 a |
| R_3_ ^b^ | 1148 ab | 482 b | 1631 ab | 523 a |
| All phenological stages (All Ps) ^b^ | 1156 a | 515 a | 1771 a | 514 a |
| **Cropping Season (CS)** |  |  |  |  |
| 2020–2021 | 1051 a | 426 a | 1478 a | 496 a |
| 2021–2022 | 1180 a | 528 a | 1708 a | 505 a |
| **ANOVA (*F probability*)** ^c^ |  |  |  |  |
| TA | 0.050 | 0.012 | 0.043 | 0.095 |
| CS | 0.680 | 0.301 | 0.329 | 0.795 |
| TA *x* CS | 0.869 | 0.297 | 0.758 | 0.279 |
| C.V. (%) | 15.0 | 15.6 | 9.67 | 6.26 |

^a^ Induction of moderate phytotoxicity by the herbicide carfentrazone-ethyl.

^b^ Application of soluble monoammonium phosphate (MAP) after induction of phytotoxicity at phenological stage V_3_.

^c^ There were no significant interactions between treatment applications or between cropping seasons.

**TABLE 3.** Soybean leaf contents of reducing sugars, sucrose, starch and RuBisCO activity as a function of the foliar application of MAP at different phenological stages, Botucatu - SP. Values in the same column with different letters are significantly different by Fisher's protected least significant difference (LSD) test at *p* ≤ 0.05. Growing season was considered a random effect.

| **Treatment Application** | **Reducing Sugars** | **Sucrose** | **Starch** | **RuBisCO** |
| --- | --- | --- | --- | --- |
|  | % | | | µmol min^−1^ mg protein^−1^ |
| Absolute control (Ac) | 2.82 a | 2.80 a | 2.57 b | 3.41 a |
| Phytotoxicity control (Pc) ^a^ | 2.47 a | 2.42 a | 3.07 a | 2.68 a |
| V_4_ ^b^ | 2.75 a | 2.72 a | 2.67 b | 2.93 a |
| V_6_ ^b^ | 2.87 a | 2.75 a | 2.55 b | 3.54 a |
| R_1_ ^b^ | 2.55 a | 2.62 a | 2.50 b | 3.00 a |
| R_3_ ^b^ | 2.80 a | 2.57 a | 2.45 b | 2.90 a |
| All phenological stages (All Ps) ^b^ | 3.02 a | 2.70 a | 2.35 b | 4.00 a |
| **Cropping Season (CS)** |  |  |  |  |
| 2020–2021 | 2.53 a | 2.56 a | 2.50 a | 3.10 a |
| 2021–2022 | 2.97 a | 2.74 a | 2.68 a | 3.32 a |
| **ANOVA (*F probability*)** ^c^ |  |  |  |  |
| TA | 0.642 | 0.937 | 0.038 | 0.058 |
| CS | 0.141 | 0.322 | 0.281 | 0.365 |
| TA *x* CS | 0.816 | 0.370 | 0.118 | 0.130 |
| C.V. (%) | 13.8 | 15.1 | 14.4 | 16.9 |

^a^ Induction of moderate phytotoxicity by the herbicide carfentrazone-ethyl.

^b^ Application of soluble monoammonium phosphate (MAP) after induction of phytotoxicity at phenological stage V_3_.

^c^ There were no significant interactions between treatment applications or between cropping seasons.

**TABLE 4.** Soybean net photosynthetic rate (*A*), stomatal conductance (*gs*), evapotranspiration (*E*), carboxylation efficiency (*A/Ci*) and water use efficiency (*WUE*) as a function of the foliar application of MAP at different phenological stages, Botucatu - SP. Values in the same column with different letters are significantly different by Fisher's protected least significant difference (LSD) test at *p* ≤ 0.05. Growing season was considered a random effect.

| **Treatment Application (TA)** | ***A*** | ***gs*** | ***E*** | ***Ci*** | ***A/Ci*** | ***WUE*** |
| --- | --- | --- | --- | --- | --- | --- |
|  | (µmol CO_2_ m^−2^ s^−1^) | mol H_2_O m^−2^ s^−1^ | mmol H_2_O m^−2^ s^−1^ | µmol CO_2_ mol^-1^ | (A/Ci) | μmol CO_2_ (mmol H_2_O)^−1^ |
| Absolute control (Ac) | 12.7 a | 225 a | 3.07 a | 199 a | 0.07 a | 4.16 a |
| Phytotoxicity control (Pc) ^a^ | 11.8 a | 210 a | 3.25 a | 211 a | 0.06 a | 3.63 a |
| V_4_ ^b^ | 11.8 a | 215 a | 3.10 a | 208 a | 0.05 a | 3.78 a |
| V_6_ ^b^ | 12.3 a | 211 a | 3.10 a | 205 a | 0.06 a | 4.01 a |
| R_1_ ^b^ | 12.4 a | 218 a | 3.00 a | 200 a | 0.06 a | 4.12 a |
| R_3_ ^b^ | 12.6 a | 218 a | 3.12 a | 202 a | 0.06 a | 4.14 a |
| All phenological stages (All Ps) ^b^ | 13.0 a | 223 a | 2.87 a | 198 a | 0.07 a | 4.53 a |
| **Cropping Season (CS)** |  |  |  |  |  |  |
| 2020–2021 | 12.3 a | 214 a | 3.47 a | 205 a | 0.06 a | 4.10 a |
| 2021–2022 | 12.4 a | 221 a | 2.63 a | 202 a | 0.06 a | 4.01 a |
| **ANOVA (*F probability*)** ^c^ |  |  |  |  |  |  |
| TA | 0.506 | 0.713 | 0.305 | 0.305 | 0.348 | 0.297 |
| CS | 0.638 | 0.209 | 0.443 | 0.443 | 0.953 | 0.660 |
| TA *x* CS | 0.596 | 0.906 | 0.124 | 0.124 | 0.529 | 0.331 |
| C.V. (%) | 10.9 | 9.3 | 6.3 | 6.3 | 12.4 | 18.0 |

^a^ Induction of moderate phytotoxicity by the herbicide carfentrazone-ethyl.

^b^ Application of soluble monoammonium phosphate (MAP) after induction of phytotoxicity at phenological stage V_3_.

^c^ There were no significant interactions between treatment applications or between cropping seasons.

**TABLE 5.** Soybean leaf contents of SOD activity, APX activity, CAT activity, H_2_O_2_, MDA and proline as a function of the foliar application of MAP at different phenological stages, Botucatu - SP. Values in the same column with different letters are significantly different by Fisher's protected least significant difference (LSD) test at *p* ≤ 0.05. Growing season was considered a random effect.

| **Treatment Application (TA)** | **SOD** | **APX** | **CAT** | **H_2_O_2_** | **MDA** | **Proline** |
| --- | --- | --- | --- | --- | --- | --- |
|  | units (U) mg^−1^ protein | nmol min^−1^ mg^−1^ protein | µmol min^−1^ mg^−1^ protein | in µmol g^−1^ | nmol g^-1^ FW | μmol g^-1^ FW |
| Absolute control (Ac) | 180 c | 11.1 b | 2.94 a | 1.32 b | 9.43 b | 0.59 a |
| Phytotoxicity control (Pc) ^a^ | 168 c | 9.01 c | 3.32 a | 1.92 a | 11.9 a | 0.62 a |
| V_4_ ^b^ | 199 a | 12.6 a | 3.36 a | 1.37 b | 9.80 b | 0.64 a |
| V_6_ ^b^ | 188 b | 10.8 b | 3.03 a | 1.53 b | 8.74 c | 0.64 a |
| R_1_ ^b^ | 186 bc | 9.83 bc | 2.76 a | 1.47 b | 8.85 c | 0.60 a |
| R_3_ ^b^ | 181 c | 10.2 bc | 3.42 a | 1.3 b | 8.90 c | 0.57 a |
| All phenological stages (All Ps) ^b^ | 196 a | 12.7 a | 3.24 a | 1.28 b | 8.56 c | 0.59 a |
| **Cropping Season (CS)** |  |  |  |  |  |  |
| 2020–2021 | 179 a | 8.11 a | 3.63 a | 1.37 a | 10.6 a | 0.72 a |
| 2021–2022 | 192 a | 14.1 a | 2.66 a | 1.54 a | 8.35 a | 0.49 a |
| **ANOVA (*F probability*)** ^c^ |  |  |  |  |  |  |
| TA | 0.046 | 0.050 | 0.921 | 0.015 | 0.006 | 0.787 |
| CS | 0.200 | 0.223 | 0.117 | 0.860 | 0.210 | 0.201 |
| TA *x* CS | 0.130 | 0.501 | 0.288 | 0.380 | 0.222 | 0.400 |
| C.V. (%) | 8.80 | 13.9 | 16.1 | 15.1 | 18.7 | 18.3 |

^a^ Induction of moderate phytotoxicity by the herbicide carfentrazone-ethyl.

^b^ Application of soluble monoammonium phosphate (MAP) after induction of phytotoxicity at phenological stage V_3_.

^c^ There were no significant interactions between treatment applications or between cropping seasons.

**TABLE 6.** Soybean population, number of branches and plant height as a function of the foliar application of MAP at different phenological stages, Botucatu - SP. Values in the same column with different letters are significantly different by Fisher's protected least significant difference (LSD) test at *p* ≤ 0.05. Growing season was considered a random effect.

| **Treatment Application (TA)** | **Population** | **Branches** | **Plant height** |
| --- | --- | --- | --- |
|  | plant ha^−1^ | Number plant^−1^ | cm |
| Absolute control (Ac) | 262750 a | 4.02 a | 88.3 a |
| Phytotoxicity control (Pc) ^a^ | 262750 a | 3.75 a | 81.8 b |
| V_4_ ^b^ | 260750 a | 3.75 a | 86.3 a |
| V_6_ ^b^ | 261250 a | 3.50 a | 86.3 a |
| R_1_ ^b^ | 262000 a | 3.55 a | 85.8 a |
| R_3_ ^b^ | 263000 a | 3.82 a | 86.5 a |
| All phenological stages (All Ps) ^b^ | 261250 a | 3.75 a | 88.3 a |
| **Cropping Season (CS)** |  |  |  |
| 2020–2021 | 255 a | 3.44 a | 85.6 a |
| 2021–2022 | 268 a | 4.04 a | 86.6 a |
| **ANOVA (*F probability*)** ^c^ |  |  |  |
| TA | 1.000 | 0.264 | 0.016 |
| CS | 0.220 | 0.410 | 0.297 |
| TA *x* CS | 0.390 | 0.302 | 0.556 |
| C.V. (%) | 7.82 | 8.75 | 4.11 |

^a^ Induction of moderate phytotoxicity by the herbicide carfentrazone-ethyl.

^b^ Application of soluble monoammonium phosphate (MAP) after induction of phytotoxicity at phenological stage V_3_.

^c^ There were no significant interactions between treatment applications or between cropping seasons.

**TABLE 7.** Soybean number of grains, weight of 100 grains (M100) and grain yield (GY) as a function of the foliar application of MAP at different phenological stages, Botucatu - SP. Values in the same column with different letters are significantly different by Fisher's protected least significant difference (LSD) test at *p* ≤ 0.05. Growing season was considered a random effect.

| **Treatment Application (TA)** | **Grains** | **M100** | **GY** |
| --- | --- | --- | --- |
|  | Number plant^−1^ | g | kg ha^−1^ |
| Absolute control (Ac) | 132 a | 18.8 a | 5515 a |
| Phytotoxicity control (Pc) ^a^ | 112 a | 18.0 a | 5203 c |
| V_4_ ^b^ | 118 a | 18.2 a | 5455 ab |
| V_6_ ^b^ | 121 a | 18.4 a | 5291 b |
| R_1_ ^b^ | 122 a | 18.5 a | 5322 b |
| R_3_ ^b^ | 121 a | 18.6 a | 5274 b |
| All phenological stages (All Ps) ^b^ | 127 a | 18.9 a | 5203 a |
| **Cropping Season (CS)** |  |  |  |
| 2020–2021 | 117 a | 18.2 a | 5572 a |
| 2021–2022 | 125 a | 18.7 a | 5172 a |
| **ANOVA (*F probability*)** ^c^ |  |  |  |
| TA | 0.069 | 0.978 | 0.029 |
| CS | 0.221 | 0.323 | 0.359 |
| TA *x* CS | 0.234 | 0.161 | 0.556 |
| C.V. (%) | 9.69 | 10.7 | 12.8 |

^a^ Induction of moderate phytotoxicity by the herbicide carfentrazone-ethyl.

^b^ Application of soluble monoammonium phosphate (MAP) after induction of phytotoxicity at phenological stage V_3_.

^c^ There were no significant interactions between treatment applications or between cropping seasons.

***MAIZE***

**TABLE 8.** Maize leaf contents of P and N as a function of the foliar application of MAP at different phenological stages, Santa Cruz do Rio Pardo - SP. Values in the same column with different letters are significantly different by Fisher's protected least significant difference (LSD) test at *p* ≤ 0.05. Growing season was considered a random effect.

| **Treatment Application (TA)** | **Phosphorus** | **Nitrogen** |
| --- | --- | --- |
|  | g kg^−1^ | |
| Absolute control (Ac) | 3.50 a | 30.8 a |
| Phytotoxicity control (Pc) ^a^ | 3.38 a | 30.9 a |
| V_4_ ^b^ | 3.40 a | 30.9 a |
| V_6_ ^b^ | 3.35 a | 29.4 a |
| V_8_ ^b^ | 3.35 a | 30.2 a |
| R_1_ ^b^ | 3.35 a | 29.1 a |
| All phenological stages (All Ps) ^b^ | 3.50 a | 31.0 a |
| **Cropping Season (CS)** |  |  |
| 2021 | 3.68 a | 30.8 a |
| 2022 | 3.22 a | 30.1 a |
| **ANOVA (*F probability*)** ^c^ |  |  |
| TA | 0.410 | 0.340 |
| CS | 0.570 | 0.460 |
| TA *x* CS | 0.310 | 0.210 |
| C.V. (%) | 8.92 | 10.2 |

^a^ Induction of moderate phytotoxicity by the herbicide carfentrazone-ethyl.

^b^ Application of soluble monoammonium phosphate (MAP) after induction of phytotoxicity at phenological stage V_3_.

^c^ There were no significant interactions between treatment applications or between cropping seasons.

**TABLE 9.** Maize leaf contents of chlorophyll *a*, chlorophyll *b*, total chlorophyll and total carotenoids as a function of the foliar application of MAP at different phenological stages, Santa Cruz do Rio Pardo - SP. Values in the same column with different letters are significantly different by Fisher's protected least significant difference (LSD) test at *p* ≤ 0.05. Growing season was considered a random effect.

| **Treatment Application (TA)** | **Chlorophyll *a*** | **Chlorophyll *b*** | **Total Chlorophyll** | **Total Carotenoids** |
| --- | --- | --- | --- | --- |
|  | mg g^−1^ fresh weight | | | |
| Absolute control (Ac) | 179 a | 108 a | 287 a | 264 a |
| Phytotoxicity control (Pc) ^a^ | 150 b | 86 c | 235 b | 206 b |
| V_4_ ^b^ | 174 a | 105 ab | 278 a | 257 a |
| V_6_ ^b^ | 171 a | 100 ab | 271 a | 261 a |
| V_8_ ^b^ | 174 a | 102 ab | 275 a | 260 a |
| R_1_ ^b^ | 173 a | 97 b | 269 a | 269 a |
| All phenological stages (All Ps) ^b^ | 177 a | 107 a | 283 a | 264 a |
| **Cropping Season (CS)** |  |  |  |  |
| 2021 | 178 a | 103 a | 276 a | 257 a |
| 2022 | 162 a | 97.0 a | 266 a | 252 a |
| **ANOVA (*F probability*)** ^c^ |  |  |  |  |
| TA | 0.001 | 0.001 | 0.001 | 0.001 |
| CS | 0.840 | 0.910 | 0.420 | 0.240 |
| TA *x* CS | 0.970 | 0.620 | 0.860 | 0.390 |
| C.V. (%) | 9.00 | 8.40 | 7.25 | 7.22 |

^a^ Induction of moderate phytotoxicity by the herbicide carfentrazone-ethyl.

^b^ Application of soluble monoammonium phosphate (MAP) after induction of phytotoxicity at phenological stage V_3_.

^c^ There were no significant interactions between treatment applications or between cropping seasons.

**TABLE 10.** Maize leaf contents of reducing sugars, total sugars and sucrose as a function of the foliar application of MAP at different phenological stages, Santa Cruz do Rio Pardo - SP. Values in the same column with different letters are significantly different by Fisher's protected least significant difference (LSD) test at *p* ≤ 0.05. Growing season was considered a random effect.

| **Treatment Application (TA)** | **Reducing Sugars** | **Total Sugars** | **Sucrose** |
| --- | --- | --- | --- |
|  | % | | |
| Absolute control (Ac) | 2.53 a | 4.65 a | 2.41 a |
| Phytotoxicity control (Pc) ^a^ | 2.55 a | 4.41 a | 1.91 b |
| V_4_ ^b^ | 2.38 a | 4.78 a | 2.47 a |
| V_6_ ^b^ | 2.47 a | 4.69 a | 2.61 a |
| V_8_ ^b^ | 2.26 a | 4.78 a | 2.48 a |
| R_1_ ^b^ | 2.37 a | 4.72 a | 2.47 a |
| All phenological stages (All Ps) ^b^ | 2.49 a | 4.78 a | 2.50 a |
| **Cropping Season (CS)** |  |  |  |
| 2021 | 2.50 | 4.39 a | 2.08 a |
| 2022 | 2.37 | 4.98 a | 2.73 a |
| **ANOVA (*F probability*)** ^c^ |  |  |  |
| TA | 0.240 | 0.770 | 0.006 |
| CS | 0.440 | 0.480 | 0.210 |
| TA *x* CS | 0.600 | 0.260 | 0.840 |
| C.V. (%) | 10.4 | 10.8 | 11.7 |

^a^ Induction of moderate phytotoxicity by the herbicide carfentrazone-ethyl.

^b^ Application of soluble monoammonium phosphate (MAP) after induction of phytotoxicity at phenological stage V_3_.

^c^ There were no significant interactions between treatment applications or between cropping seasons.

**TABLE 11.** Maize leaf contents of starch and RuBisCO activity as a function of the foliar application of MAP at different phenological stages, Santa Cruz do Rio Pardo - SP. Values in the same column with different letters are significantly different by Fisher's protected least significant difference (LSD) test at *p* ≤ 0.05. Growing season was considered a random effect.

| **Treatment Application** (TA) | **Starch** | **RuBisCO** |
| --- | --- | --- |
|  | % | µmol min^−1^ mg protein^−1^ |
| Absolute control (Ac) | 2.04 b | 55.5 a |
| Phytotoxicity control (Pc) ^a^ | 2.60 a | 47.3 b |
| V_4_ ^b^ | 2.22 b | 55.8 a |
| V_6_ ^b^ | 2.02 b | 55.8 a |
| V_8_ ^b^ | 1.96 b | 53.9 a |
| R_1_ ^b^ | 1.93 b | 56.1 a |
| All phenological stages (All Ps) ^b^ | 1.92 b | 56.7 a |
| **Cropping Season (CS)** |  |  |
| 2021 | 2.17 a | 52.7 a |
| 2022 | 2.03 a | 56.1 a |
| **ANOVA (*F probability*)** ^c^ |  |  |
| TA | 0.001 | 0.001 |
| CS | 0.490 | 0.850 |
| TA *x* CS | 0.970 | 0.800 |
| C.V. (%) | 12.9 | 8.00 |

^a^ Induction of moderate phytotoxicity by the herbicide carfentrazone-ethyl.

^b^ Application of soluble monoammonium phosphate (MAP) after induction of phytotoxicity at phenological stage V_3_.

^c^ There were no significant interactions between treatment applications or between cropping seasons.

**TABLE 12.** Maize leaf net photosynthetic rate (*A*), stomatal conductance (*gs*), evapotranspiration (*E*), carboxylation efficiency (*A/Ci*) and water use efficiency (WUE) as a function of the foliar application of MAP at different phenological stages, Santa Cruz do Rio Pardo - SP. Values in the same column with different letters are significantly different by Fisher's protected least significant difference (LSD) test at *p* ≤ 0.05. Growing season was considered a random effect.

| **Treatment Application (TA)** | ***A*** | ***gs*** | ***E*** | ***Ci*** | ***A/Ci*** | ***WUE*** |
| --- | --- | --- | --- | --- | --- | --- |
|  | (µmol CO_2_ m^−2^ s^−1^) | mol H_2_O m^−2^ s^−1^ | mmol H_2_O m^−2^ s^−1^ | µmol CO_2_ mol^−1^ | (A/Ci) | μmol CO_2_ (mmol H_2_O)^−1^ |
| Absolute control (Ac) | 23.1 a | 190 a | 3.52 c | 137 a | 0.19 ab | 7.70 a |
| Phytotoxicity control (Pc) ^a^ | 19.9 b | 169 b | 4.15 a | 147 a | 0.13 c | 5.87 b |
| V_4_ ^b^ | 23.0 a | 193 a | 3.80 b | 123 b | 0.18 b | 7.33 a |
| V_6_ ^b^ | 22.7 a | 191 a | 3.65 bc | 120 b | 0.19 ab | 7.49 a |
| V_8_ ^b^ | 22.8 a | 192 a | 3.52 c | 119 b | 0.19 ab | 7.48 a |
| R_1_ ^b^ | 23.1 a | 192 a | 3.45 c | 120 b | 0.19 ab | 7.83 a |
| All phenological stages (All Ps) ^b^ | 23.4 a | 193 a | 3.49 c | 115 b | 0.20 a | 7.43 a |
| **Cropping Season (CS)** |  |  |  |  |  |  |
| 2021 | 24.8 a | 191 a | 3.38 a | 125 a | 0.19 a | 8.40 a |
| 2022 | 20.3 a | 186 a | 3.93 a | 127 a | 0.17 a | 6.20 a |
| **ANOVA (*F probability*)** ^c^ |  |  |  |  |  |  |
| TA | 0.001 | 0.001 | 0.001 | 0.001 | 0.001 | 0.001 |
| Year | 0.320 | 0.760 | 0.130 | 0.370 | 0.600 | 0.500 |
| T x Y | 0.620 | 0.920 | 0.540 | 0.220 | 0.250 | 0.230 |
| C.V. | 5.23 | 7.25 | 7.27 | 7.11 | 11.1 | 9.57 |

^a^ Induction of moderate phytotoxicity by the herbicide carfentrazone-ethyl.

^b^ Application of soluble monoammonium phosphate (MAP) after induction of phytotoxicity at phenological stage V_3_.

^c^ There were no significant interactions between treatment applications or between cropping seasons.

**TABLE 13.** Maize leaf contents of SOD activity, APX activity, CAT activity, H_2_O_2_, MDA and proline as a function of the foliar application of MAP at different phenological stages, Santa Cruz do Rio Pardo - SP. Values in the same column with different letters are significantly different by Fisher's protected least significant difference (LSD) test at *p* ≤ 0.05. Growing season was considered a random effect.

| **Treatment Application (TA)** | **SOD** | **APX** | **CAT** | **H_2_O_2_** | **MDA** | **Proline** |
| --- | --- | --- | --- | --- | --- | --- |
|  | units (U) mg^−1^ protein | nmol min^−1^ mg^−1^ protein | µmol min^−1^ mg^−1^ protein | in µmol g^−1^ | nmol g^-1^ FW | µmol min^−1^ mg^−1^ protein |
| Absolute control (Ac) | 169 b | 41.8 a | 1.11 a | 4.70 ab | 8.90 b | 1.24 a |
| Phytotoxicity control (Pc) ^a^ | 148 d | 32.2 a | 0.85 a | 5.47 a | 11.4 a | 1.31 a |
| V_4_ ^b^ | 171 b | 38.2 a | 1.05 a | 4.72 ab | 9.80 b | 1.26 a |
| V_6_ ^b^ | 172 b | 37.2 a | 1.01 a | 4.37 b | 8.74 b | 1.27 a |
| V_8_ ^b^ | 160 c | 36.7 a | 0.99 a | 4.26 b | 8.85 b | 1.22 a |
| R_1_ ^b^ | 158 c | 35.8 a | 0.97 a | 4.25 b | 8.90 b | 1.17 a |
| All phenological stages (All Ps) ^b^ | 182 a | 41.7 a | 1.03 a | 3.95 b | 8.81 b | 1.13 a |
| **Cropping Season (CS)** |  |  |  |  |  |  |
| 2021 | 164 a | 38.7 a | 0.93 a | 4.37 a | 9.18 a | 1.27 a |
| 2022 | 167 a | 36.5 a | 1.07 a | 4.69 a | 9.67 a | 1.18 a |
| **ANOVA (*F probability*)** ^c^ |  |  |  |  |  |  |
| TA | 0.001 | 0.080 | 0.460 | 0.001 | 0.030 | 0.490 |
| Year | 0.780 | 0.190 | 0.300 | 0.190 | 0.470 | 0.510 |
| T x Y | 0.530 | 0.410 | 0.800 | 0.350 | 0.810 | 0.660 |
| C.V. | 4.57 | 13.7 | 12.9 | 16.8 | 14.5 | 14.7 |

^a^ Induction of moderate phytotoxicity by the herbicide carfentrazone-ethyl.

^b^ Application of soluble monoammonium phosphate (MAP) after induction of phytotoxicity at phenological stage V_3_.

^c^ There were no significant interactions between treatment applications or between cropping seasons.

**TABLE 14.** Maize final population, plant height and number of rows as a function of the foliar application of MAP at different phenological stages, Santa Cruz do Rio Pardo - SP. Values in the same column with different letters are significantly different by Fisher's protected least significant difference (LSD) test at *p* ≤ 0.05. Growing season was considered a random effect.

| **Treatment Application (TA)** | **Final Population** | **Plant height** | **Rows** |
| --- | --- | --- | --- |
|  | plant ha^−1^ | cm | Number ear^−1^ |
| Absolute control (Ac) | 57889 a | 175 a | 16.6 a |
| Phytotoxicity control (Pc) ^a^ | 57314 a | 170 b | 15.8 a |
| V_4_ ^b^ | 57649 a | 171 a | 16.0 a |
| V_6_ ^b^ | 57013 a | 175 a | 16.3 a |
| V_8_ ^b^ | 57190 a | 170 a | 16.2 a |
| R_1_ ^b^ | 57506 a | 174 a | 16.3 a |
| All phenological stages (All Ps) ^b^ | 57475 a | 177 a | 16.4 a |
| **Cropping Season (CS)** |  |  |  |
| 2021 | 58444 a | 186 a | 16.3 a |
| 2022 | 56424 a | 161 a | 16.2 a |
| **ANOVA (*F probability*)** ^c^ |  |  |  |
| TA | 0.700 | 0.120 | 0.700 |
| Year | 0.610 | 0.190 | 0.980 |
| T x Y | 0.790 | 0.210 | 0.990 |
| C.V. | 10.8 | 3.43 | 5.76 |

^a^ Induction of moderate phytotoxicity by the herbicide carfentrazone-ethyl.

^b^ Application of soluble monoammonium phosphate (MAP) after induction of phytotoxicity at phenological stage V_3_.

^c^ There were no significant interactions between treatment applications or between cropping seasons.

**TABLE 15.** Maize number of grains, weight of 100 grains (M100) and grain yield (GY) as a function of the foliar application of MAP at different phenological stages, Santa Cruz do Rio Pardo - SP. Values in the same column with different letters are significantly different by Fisher's protected least significant difference (LSD) test at *p* ≤ 0.05. Growing season was considered a random effect.

| **Treatment Application (TA)** | **Grains** | **M100** | **GY** |
| --- | --- | --- | --- |
|  | Number row^−1^ | g | kg ha^−1^ |
| Absolute control (Ac) | 27.6 a | 20.5 a | 5880 a |
| Phytotoxicity control (Pc) ^a^ | 26.3 a | 18.0 a | 5269 c |
| V_4_ ^b^ | 27.1 a | 20.3 a | 5568 b |
| V_6_ ^b^ | 26.9 a | 20.0 a | 5625 b |
| V_8_ ^b^ | 26.8 a | 20.7 a | 5569 b |
| R_1_ ^b^ | 27.0 a | 20.3 a | 5575 b |
| All phenological stages (All Ps) ^b^ | 27.5 a | 20.9 a | 5902 a |
| **Cropping Season (CS)** |  |  |  |
| 2021 | 27.8 a | 19.9 a | 5690 a |
| 2022 | 26.3 a | 20.7 a | 5563 a |
| **ANOVA (*F probability*)** ^c^ |  |  |  |
| TA | 0.420 | 0.060 | 0.010 |
| Year | 0.320 | 0.830 | 0.130 |
| T x Y | 0.910 | 0.620 | 0.980 |
| C.V. | 2.95 | 11.0 | 3.78 |

^a^ Induction of moderate phytotoxicity by the herbicide carfentrazone-ethyl.

^b^ Application of soluble monoammonium phosphate (MAP) after induction of phytotoxicity at phenological stage V_3_.

^c^ There were no significant interactions between treatment applications or between cropping seasons.

***COTTON***

**TABLE 16.** Cotton leaf content of P and N as a function of the foliar application of MAP at different phenological stages, Riolândia - SP. Values in the same column with different letters are significantly different by Fisher's protected least significant difference (LSD) test at *p* ≤ 0.05. Growing season was considered a random effect.

| **Treatment Application (TA)** | **Phosphorus** | **Nitrogen** |
| --- | --- | --- |
|  | g kg^−1^ | |
| Absolute control (Ac) | 1.85 a | 37.5 a |
| Phytotoxicity control (Pc) ^a^ | 1.77 a | 37.2 a |
| B_1_ ^b^ | 1.87 a | 37.7 a |
| F_1_ ^b^ | 1.87 a | 36.6 a |
| C_1_ ^b^ | 1.90 a | 37.9 a |
| C_4_ ^b^ | 1.87 a | 36.9 a |
| All phenological stages (All Ps) ^b^ | 1.87 a | 37.8 a |
| **Cropping Season (CS)** |  |  |
| 2020–2021 | 1.81 a | 37.4 a |
| 2021–2022 | 1.75 a | 35.9 a |
| **ANOVA (*F probability*)** ^c^ |  |  |
| TA | 0.520 | 0.640 |
| Year | 0.260 | 0.960 |
| T x Y | 0.730 | 0.250 |
| C.V. | 8.71 | 7.83 |

^a^ Induction of moderate phytotoxicity by the herbicide carfentrazone-ethyl.

^b^ Application of soluble monoammonium phosphate (MAP) after induction of phytotoxicity at phenological stage V_4_.

^c^ There were no significant interactions between treatment applications or between cropping seasons

**TABLE 17.** Cotton leaf contents of chlorophyll *a*, chlorophyll *b*, total chlorophyll and total carotenoids as a function of the foliar application of MAP at different phenological stages, Riolândia - SP. Values in the same column with different letters are significantly different by Fisher's protected least significant difference (LSD) test at *p* ≤ 0.05. Growing season was considered a random effect.

| **Treatment Application (TA)** | **Chlorophyll *a*** | **Chlorophyll *b*** | **Total Chlorophyll** | **Total Carotenoids** |
| --- | --- | --- | --- | --- |
|  | mg g^−1^ fresh weight | | | |
| Absolute control (Ac) | 1508 a | 444 a | 1952 a | 429 a |
| Phytotoxicity control (Pc) ^a^ | 1294 c | 396 c | 1690 d | 378 c |
| B_1_ ^b^ | 1489 a | 429 ab | 1919 a | 401 b |
| F_1_ ^b^ | 1376 b | 417 b | 1793 c | 394 b |
| C_1_ ^b^ | 1422 b | 422 b | 1845 b | 390 b |
| C_4_ ^b^ | 1430 b | 419 b | 1850 b | 402 b |
| All phenological stages (All Ps) ^b^ | 1484 a | 423 b | 1980 a | 416 ab |
| **Cropping Season (CS)** |  |  |  |  |
| 2020–2021 | 1447 a | 402 a | 1873 a | 384 a |
| 2021–2022 | 1411 a | 441 a | 1830 a | 419 a |
| **ANOVA (*F probability*)** ^c^ |  |  |  |  |
| TA | 0.001 | 0.001 | 0.001 | 0.001 |
| Year | 0.490 | 0.330 | 0.520 | 0.120 |
| T x Y | 0.170 | 0.570 | 0.440 | 0.830 |
| C.V. | 3.17 | 3.83 | 5.69 | 5.73 |

^a^ Induction of moderate phytotoxicity by the herbicide carfentrazone-ethyl.

^b^ Application of soluble monoammonium phosphate (MAP) after induction of phytotoxicity at phenological stage V_4_.

^c^ There were no significant interactions between treatment applications or between cropping seasons

**TABLE 18.** Cotton leaf contents of reducing sugars, total sugars and sucrose as a function of the foliar application of MAP at different phenological stages, Riolândia - SP. Values in the same column with different letters are significantly different by Fisher's protected least significant difference (LSD) test at *p* ≤ 0.05. Growing season was considered a random effect.

| **Treatment Application (TA)** | **Reducing Sugars** | **Total Sugars** | **Sucrose** |
| --- | --- | --- | --- |
|  | % | | |
| Absolute control (Ac) | 2.98 a | 5.67 a | 2.94 a |
| Phytotoxicity control (Pc) ^a^ | 3.23 a | 5.61 a | 2.27 b |
| B_1_ ^b^ | 2.82 a | 5.68 a | 2.66 a |
| F_1_ ^b^ | 2.75 a | 5.71 a | 2.53 a |
| C_1_ ^b^ | 2.93 a | 5.93 a | 2.45 a |
| C_4_ ^b^ | 2.85 a | 5.85 a | 2.63 a |
| All phenological stages (All Ps) ^b^ | 2.75 a | 6.03 a | 3.14 a |
| **Cropping Season (CS)** |  |  |  |
| 2020–2021 | 2.64 a | 5.11 a | 2.63 a |
| 2021–2022 | 3.17 a | 6.45 a | 2.69 a |
| **ANOVA (*F probability*)** ^c^ |  |  |  |
| TA | 0.360 | 0.860 | 0.001 |
| Year | 0.270 | 0.540 | 0.310 |
| T x Y | 0.940 | 0.980 | 0.510 |
| C.V. | 14.9 | 11.7 | 13.8 |

^a^ Induction of moderate phytotoxicity by the herbicide carfentrazone-ethyl.

^b^ Application of soluble monoammonium phosphate (MAP) after induction of phytotoxicity at phenological stage V_4_ .

^c^ There were no significant interactions between treatment applications or between cropping seasons.

**TABLE 19.** Cotton leaf contents of starch and RuBisCO activity as a function of the foliar application of MAP at different phenological stages, Riolândia - SP. Values in the same column with different letters are significantly different by Fisher's protected least significant difference (LSD) test at *p* ≤ 0.05. Growing season was considered a random effect.

| **Treatment Application (TA)** | **Starch** | **RuBisCO** |
| --- | --- | --- |
|  | % | µmol min^−1^ mg protein^−1^ |
| Absolute control (Ac) | 2.92 b | 20.7 ab |
| Phytotoxicity control (Pc) ^a^ | 3.50 a | 18.5 c |
| B_1_ ^b^ | 2.70 bc | 20.0 bc |
| F_1_ ^b^ | 2.53 c | 20.3 bc |
| C_1_ ^b^ | 2.51 c | 20.8 c |
| C_4_ ^b^ | 2.63 bc | 20.5 bc |
| All phenological stages (All Ps) ^b^ | 2.34 c | 21.5 a |
| **Cropping Season (CS)** |  |  |
| 2020–2021 | 2.33 a | 19.9 a |
| 2021–2022 | 3.13 a | 20.7 a |
| **ANOVA (*F probability*)** ^c^ |  |  |
| TA | 0.001 | 0.001 |
| Year | 0.600 | 0.340 |
| T x Y | 0.590 | 0.140 |
| C.V. | 13.7 | 4.77 |

^a^ Induction of moderate phytotoxicity by the herbicide carfentrazone-ethyl.

^b^ Application of soluble monoammonium phosphate (MAP) after induction of phytotoxicity at phenological stage V_4_.

^c^ There were no significant interactions between treatment applications or between cropping seasons.

**TABLE 20.** Cotton leaf net photosynthetic rate (*A*), stomatal conductance (*gs*), evapotranspiration (*E*), carboxylation efficiency (*A/Ci*) and water use efficiency (WUE) as a function of the foliar application of MAP at different phenological stages, Riolândia - SP. Values in the same column with different letters are significantly different by Fisher's protected least significant difference (LSD) test at *p* ≤ 0.05. Growing season was considered a random effect.

| **Treatment Application (TA)** | ***A*** | ***gs*** | ***E*** | ***Ci*** | ***A/Ci*** | ***WUE*** |
| --- | --- | --- | --- | --- | --- | --- |
|  | (µmol CO_2_ m^−2^ s^−1^) | mol H_2_O m^−2^ s^−1^ | mmol H_2_O m^−2^ s^−1^ | µmol CO_2_ mol^−1^ | Adimensional | μmol CO_2_ (mmol H_2_O)^−1^ |
| Absolute control (Ac) | 17.4 b | 203 b | 3.21 a | 197 b | 5.29 b | 0.12 b |
| Phytotoxicity control (Pc) ^a^ | 14.7 d | 218 d | 3.27 a | 156 a | 4.55 c | 0.09 c |
| B_1_ ^b^ | 15.8 c | 197 c | 3.19 a | 185 b | 5.10 b | 0.12 b |
| F_1_ ^b^ | 16.6 bc | 196 c | 3.17 a | 180 b | 5.36 b | 0.12 b |
| C_1_ ^b^ | 16.9 b | 196 b | 3.14 a | 205 b | 5.54 b | 0.12 b |
| C_4_ ^b^ | 16.9 b | 197 b | 3.13 a | 201 b | 5.52 b | 0.12 b |
| All phenological stages (All Ps) ^b^ | 18.6 a | 195 a | 3.08 a | 227 b | 6.17 a | 0.13 a |
| **Cropping Season (CS)** |  |  |  |  |  |  |
| 2020–2021 | 17.8 a | 214 a | 3.28 a | 195 a | 5.02 a | 0.11 a |
| 2021–2022 | 15.7 a | 185 a | 3.17 a | 191 a | 5.71 a | 0.12 a |
| **ANOVA (*F probability*)** ^c^ |  |  |  |  |  |  |
| TA | 0.001 | 0.001 | 0.540 | 0.002 | 0.001 | 0.001 |
| Year | 0.660 | 0.120 | 0.930 | 0.270 | 0.760 | 0.300 |
| T x Y | 0.130 | 0.400 | 0.940 | 0.150 | 0.540 | 0.810 |
| C.V. | 4.66 | 6.68 | 5.72 | 7.23 | 7.98 | 8.32 |

^a^ Induction of moderate phytotoxicity by the herbicide carfentrazone-ethyl.

^b^ Application of soluble monoammonium phosphate (MAP) after induction of phytotoxicity at phenological stage V_4_.

^c^ There were no significant interactions between treatment applications or between cropping seasons.

**TABLE 21.** Cotton leaf contents of SOD activity, APX activity, CAT activity, H_2_O_2_, MDA and proline as a function of the foliar application of MAP at different phenological stages, Riolândia - SP. Values in the same column with different letters are significantly different by Fisher's protected least significant difference (LSD) test at *p* ≤ 0.05. Growing season was considered a random effect.

| **Treatment Application (TA)** | **SOD** | **APX** | **CAT** | **H_2_O_2_** | **MDA** | **Proline** |
| --- | --- | --- | --- | --- | --- | --- |
|  | units (U) mg^−1^ protein | nmol min^−1^ mg^−1^ protein | µmol min^−1^ mg^−1^ protein | in µmol g^−1^ | nmol g^-1^ FW | µmol min^−1^ mg^−1^ protein |
| Absolute control (Ac) | 175 a | 51.3 a | 4.89 a | 11.2 c | 26.2 b | 1.54 a |
| Phytotoxicity control (Pc) ^a^ | 167 a | 49.5 a | 4.86 a | 13.7 a | 29.6 a | 1.54 a |
| B_1_ ^b^ | 180 a | 52.6 a | 4.72 a | 12.1 b | 25.4 b | 1.68 a |
| F_1_ ^b^ | 181 a | 50.9 a | 4.91 a | 12.2 b | 25.8 b | 1.66 a |
| C_1_ ^b^ | 181 a | 51.4 a | 4.90 a | 12.4 b | 24.9 b | 1.63 a |
| C_4_ ^b^ | 175 a | 50.5 a | 4.94 a | 11.9 b | 24.8 b | 1.57 a |
| All phenological stages (All Ps) ^b^ | 184 a | 53.4 a | 5.01 a | 12.0 b | 25.0 b | 1.63 a |
| **Cropping Season (CS)** |  |  |  |  |  |  |
| 2020–2021 | 171 a | 52.2 a | 5.43 a | 11.6 a | 25.0 a | 1.51 a |
| 2021–2022 | 185 a | 50.5 a | 4.34 a | 12.8 a | 26.0 a | 1.70 a |
| **ANOVA (*F probability*)** ^c^ |  |  |  |  |  |  |
| TA | 0.410 | 0.970 | 0.690 | 0.010 | 0.020 | 0.960 |
| Year | 0.170 | 0.480 | 0.540 | 0.180 | 0.390 | 0.870 |
| T x Y | 0.330 | 0.740 | 0.460 | 0.580 | 0.760 | 0.410 |
| C.V. | 6.85 | 5.82 | 6.28 | 5.16 | 9.05 | 8.42 |

^a^ Induction of moderate phytotoxicity by the herbicide carfentrazone-ethyl.

^b^ Application of soluble monoammonium phosphate (MAP) after induction of phytotoxicity at phenological stage V_4_.

^c^ There were no significant interactions between treatment applications or between cropping seasons.

**TABLE 22.** Cotton population, plant height and number of branches as a function of the foliar application of MAP at different phenological stages, Riolândia - SP. Values in the same column with different letters are significantly different by Fisher's protected least significant difference (LSD) test at *p* ≤ 0.05. Growing season was considered a random effect.

| **Treatment Application (TA)** | **Final Population** | **Plant height** | **Branches** |
| --- | --- | --- | --- |
|  | plant ha^−1^ x 1000 | cm | Number plant^−1^ |
| Absolute control (Ac) | 96.9 a | 86.1 a | 8.86 bc |
| Phytotoxicity control (Pc) ^a^ | 96.5 a | 77.3 b | 8.37 c |
| B_1_ ^b^ | 96.3 a | 84.8 a | 8.84 bc |
| F_1_ ^b^ | 94.3 a | 87.6 a | 8.94 bc |
| C_1_ ^b^ | 96.4 a | 84.5 a | 8.62 bc |
| C_4_ ^b^ | 95.5 a | 86.2 a | 9.02 b |
| All phenological stages (All Ps) ^b^ | 94.9 a | 86.6 a | 10.1 a |
| **Cropping Season (CS)** |  |  |  |
| 2020–2021 | 101 a | 84.8 a | 8.26 a |
| 2021–2022 | 90.0 a | 84.7 a | 9.66 a |
| **ANOVA (*F probability*)** ^c^ |  |  |  |
| TA | 0.960 | 0.050 | 0.001 |
| Year | 0.470 | 0.930 | 0.870 |
| T x Y | 0.810 | 0.430 | 0.250 |
| C.V. | 6.54 | 5.91 | 6.48 |

^a^ Induction of moderate phytotoxicity by the herbicide carfentrazone-ethyl.

^b^ Application of soluble monoammonium phosphate (MAP) after induction of phytotoxicity at phenological stage V_4_.

^c^ There were no significant interactions between treatment applications or between cropping seasons.

**TABLE 23.** Cotton number of bolls, boll weight and fiber yield as a function of the foliar application of MAP at different phenological stages, Riolândia - SP. Values in the same column with different letters are significantly different by Fisher's protected least significant difference (LSD) test at *p* ≤ 0.05. Growing season was considered a random effect.

| **Treatment Application (TA)** | **Bolls** | **Boll weight** | **Cotton fiber yield** |
| --- | --- | --- | --- |
|  | Number | g | kg ha^-1^ |
| Absolute control (Ac) | 10.8 a | 4.44 a | 3731 a |
| Phytotoxicity control (Pc) ^a^ | 9.51 a | 3.68 c | 2798 c |
| B_1_ ^b^ | 10.0 a | 4.14 ab | 3633 ab |
| F_1_ ^b^ | 10.3 a | 3.95 b | 3541 b |
| C_1_ ^b^ | 10.2 a | 4.04 b | 3538 b |
| C_4_ ^b^ | 10.4 a | 4.00 b | 3536 b |
| All phenological stages (All Ps) ^b^ | 10.9 a | 4.38 b | 3701 a |
| **Cropping Season (CS)** |  |  |  |
| 2020–2021 | 10.1 a | 4.54 a | 1573 a |
| 2021–2022 | 10.4 a | 3.65 a | 1603 a |
| **ANOVA (*F probability*)** ^c^ |  |  |  |
| TA | 0.280 | 0.010 | 0.010 |
| Year | 0.840 | 0.750 | 0.580 |
| T x Y | 0.560 | 0.390 | 0.690 |
| C.V. | 8.07 | 4.81 | 12.8 |

^a^ Induction of moderate phytotoxicity by the herbicide carfentrazone-ethyl.

^b^ Application of soluble monoammonium phosphate (MAP) after induction of phytotoxicity at phenological stage V_4_.

^c^ There were no significant interactions between treatment applications or between cropping seasons.
